# Epigenetic mutation load is weakly correlated with epigenetic age acceleration

**DOI:** 10.18632/aging.103950

**Published:** 2020-09-29

**Authors:** Qi Yan, Kimberly C. Paul, Ake T. Lu, Cynthia Kusters, Alexandra M. Binder, Steve Horvath, Beate Ritz

**Affiliations:** 1 Department of Epidemiology, UCLA Fielding School of Public Health, Los Angeles, CA 90095, USA; 2 Department of Human Genetics, David Geffen School of Medicine, University of California Los Angeles, Los Angeles, CA 90095, USA; 3 Population Sciences in the Pacific Program (Cancer Epidemiology), University of Hawaii Cancer Center, University of Hawaii at Manoa, Honolulu, HI 96813, USA; 4 Department of Biostatistics, Fielding School of Public Health, University of California Los Angeles, Los Angeles, CA 90095, USA; 5 Department of Neurology, UCLA School of Medicine, Los Angeles, CA 90095, USA

**Keywords:** stochastic epigenetic mutation, epigenetic mutation load, aging, epigenetic clock, DNA methylation

## Abstract

DNA methylation (DNAm) age estimators are widely used to study aging-related conditions. It is not yet known whether DNAm age is associated with the accumulation of stochastic epigenetic mutations (SEMs), which reflect dysfunctions of the epigenetic maintenance system. Here, we defined epigenetic mutation load (EML) as the total number of SEMs per individual. We assessed associations between EML and DNAm age acceleration estimators using biweight midcorrelations in four population-based studies (total n = 6,388). EML was not only positively associated with chronological age (meta r = 0.171), but also with four measures of epigenetic age acceleration: the Horvath pan tissue clock, intrinsic epigenetic age acceleration, the Hannum clock, and the GrimAge clock (meta-analysis correlation ranging from r = 0.109 to 0.179). We further conducted pathway enrichment analyses for each participant’s SEMs. The enrichment result demonstrated the stochasticity of epigenetic mutations, meanwhile implicated several pathways: signaling, neurogenesis, neurotransmitter, glucocorticoid, and circadian rhythm pathways may contribute to faster DNAm age acceleration. Finally, investigating genomic-region specific EML, we found that EMLs located within regions of transcriptional repression (TSS1500, TSS200, and 1stExon) were associated with faster age acceleration. Overall, our findings suggest a role for the accumulation of epigenetic mutations in the aging process.

## INTRODUCTION

Epigenetic changes are an important hallmark of aging [1–3]. DNA methylation analysis provided promising molecular biomarkers of aging [4], with several epigenetic aging clocks having been introduced and used by aging researchers in recent years [5–12]. Age-adjusted epigenetic age estimates (referred to as epigenetic age acceleration) have been linked to a large number of age-related conditions [6, 7, 13–20].

Here we set out to investigate whether DNAm clocks possibly capture any dysfunction of the epigenetic maintenance system (EMS) of a cell [5, 13, 21]. Age is known to greatly increase the variability of DNA methylation levels and the epigenetic profiles of monozygotic twins diverge considerably with age [22, 23]. Gentilini et al [24] proposed that stochastic epigenetic mutations (SEMs) increase exponentially with chronological age. The association of SEMs and aging was for the first time longitudinally assessed in the Swedish twin cohort [25] which confirmed that epigenetic mutations accumulate with age in an individual. In addition, SEMs have recently been associated with hepatocellular carcinoma staging [26], exposure to endocrine-disrupting compounds [27], socioeconomic position, and lifestyle factors [28]. Despite extensive research in this field, to our knowledge, most previous studies focused on chronological age rather than epigenetic age and epigenetic age acceleration. One study found SEM counts to be positively associated with epigenetic age acceleration based on both the Horvath and Hannum clocks [27]. Another recent study focused on Hannum, GrimAge, and intrinsic epigenetic age estimators within the Generation Scotland and the Lothian Birth Cohort, and reported positive associations between SEM counts and all three epigenetic age measurements [29]. To address the complexity of the aging process and the biological mechanisms underlying different epigenetic clocks, it may be useful to systematically study multiple clocks at the same time. In addition, biologic pathway enrichment analysis may help us gain an understanding of the pathophysiology of accelerated aging.

We pooled four population-based studies (total n = 6,388) to systematically investigate whether SEM counts are associated with epigenetic age acceleration. We included four DNAm aging clocks that represent different manifestations of the epigenetic aging processes, including: the pan-tissue chronological age estimator by Horvath (2013, Horvath clock) [5]; an intrinsic epigenetic age measure derived from the Horvath clock by additionally regressing out cell compositions (intrinsic clock) [30]; the leukocyte-based chronological age estimator by Hannum et al. (2013, Hannum clock) [11]; and the epigenetic mortality risk predictor developed recently by Lu et al. (2019, GrimAge clock) [7]. Age-adjusted versions of these biomarkers are generally being referred to as measures of epigenetic age acceleration and denoted as AgeAccelHorvath, intrinsic epigenetic age acceleration (IEAA), AgeAccelHannum, and AgeAccelGrim, respectively. We also coined the new term “epigenetic mutation load (EML)” as representing the total number of SEMs observed for each individual. In this article, we will 1) relate EML to different epigenetic age acceleration measures; 2) functionally annotate mutated CpG sites; 3) conduct biological pathway enrichment analysis; 4) relate DNA region-specific EMLs to epigenetic measures of age acceleration; and 5) compare SEMs with the Shannon entropy measure as the latter can be interpreted as alternative measure for the decline of epigenetic maintenance.

## RESULTS

### Study population demographics

Our study includes 6,388 individuals from 4 studies: the Framingham Heart Study (FHS) Offspring Cohort, the Women’s Health Initiative (WHI), the Jackson Heart Study (JHS), and the Parkinson’s Environment and Genes (wave 1) known as the PEG1 study.

The main characteristics of the study populations are shown in Table 1. Briefly, FHS provided data for 2,326 individuals, with nearly half of them male (n = 1077; 46%) and all are white. Of the 2,091 female participants from the WHI, 989 (47%) are non-Hispanic white, 431 (21%) Hispanic, and 671 (32%) African American. JHS investigated 1,734 African American individuals with a majority of female participants (n = 1086; 63%). The 237 PEG1 control study participants were mostly non-Hispanic white (n = 207; 87%), and half were male (n = 126; 53%). The age ranges varied with the JHS having the largest range (22-93; mean = 56.2), and WHI the smallest (50-80; mean = 65.4). Mean ages of all populations ranged between 56.2 and 67.4. Additional details on cohorts and participant characteristics can be found in the Methods.

**Table 1:** Distribution of demographics and DNAm aging clocks.

	**FHS (n = 2326)**	**WHI (n= 2091)**	**JHS (n= 1734)**	**PEG 1 (n = 237)**
**Age**				
Min	40	50	22	35
Max	92	80	93	92
Mean (SD)	66.36 (8.94)	65.34 (7.10)	56.21 (12.30)	67.42 (12.82)
**Sex**				
Male (%)	1,077 (46)	0 (0)	648 (37)	126 (53)
Female (%)	1,249 (54)	2,091 (100)	1,086 (63)	111 (47)
**Race/Ethnicity**				
White (%)	2,326 (100)	989(47)	0 (0)	207 (87)
Hispanic (%)	0 (0)	431 (21)	0 (0)	19 (8)
African American (%)	0 (0)	671 (32)	1734 (100)	0 (0)
Native American (%)	0 (0)	0 (0)	0 (0)	11 (5)
**AgeAccelHorvath**				
Min	-16.03	-22.56	-16.57	-13.44
Median	-0.38	-0.07	-0.07	-0.13
Max	41.62	29.35	22.81	22.98
Mean (SD)	-0.08 (4.81)	0.10 (5.18)	0.04 (4.45)	0.00 (5.31)
**IEAA**				
Min	-21.83	-21.46	-15.67	-12.17
Median	-0.17	-0.05	0.07	-0.13
Max	26.93	24.89	22.40	20.28
Mean (SD)	-0.03 (4.59)	0.02 (4.88)	0.05 (4.34)	0.00 (4.92)
**AgeAccelHannum**				
Min	-19.25	-19.50	-11.59	-12.92
Median	-0.18	0.02	-0.15	-0.27
Max	27.97	18.19	19.35	12.53
Mean (SD)	-0.02 (4.83)	0.02 (4.80)	0.03 (3.49)	0.00 (4.42)
**AgeAccelGrim**				
Min	-10.92	-10.03	-13.66	-8.74
Median	-0.76	-0.47	-0.81	-0.64
Max	22.51	16.35	24.94	14.62
Mean (SD)	0.02 (4.86)	0.01 (3.80)	0.01 (4.81)	0.00 (4.50)

### Epigenetic mutation load is the number of SEMs

All DNA methylation data was extracted from blood samples with the Illumina Infinium platform (450K array for PEG1, FHS, and WHI studies; EPIC array for WHI). Following a published and validated approach [24, 26, 31], a SEM is observed for a given person at a specific CpG site if an individual’s methylation level is more than three times the interquartile range (IQR) lower than the 25^th^ percentile (Q1 – 3 × IQR), or more than three times the IQR higher than the 75^th^ percentile (Q3 + 3 × IQR). The 25^th^ and 75^th^ percentile, and correspondingly the IQR, for each CpG locus was estimated across all samples. Furthermore, we defined the epigenetic mutation load (EML) of each study participant according to the total number of SEMs.

EML was highly variable across people (Supplementary Table 1), with a mean value ranging from 1647 to 3401 depending on the total number of CpGs measured on different arrays (FHS: 2433; WHI: 1647; JHS: 3401; PEG1: 2137). Since EMLs were not normally distributed, natural log-transformed EML values were used in all analyses.

EML was not associated with microarray slides (ANOVA *p* = 0.135) or position on the array (ANOVA *p* = 0.458). Also, EML was not correlated with the average intensity of bisulfite conversion controls (Pearson r = -0.085, p = 0.194). Thus, we concluded that the EML was independent of batches or other technical aspects.

### Correlations among DNAm aging clocks

We calculated all DNAm aging estimators including the Horvath clock, the Hannum clock, the GrimAge clock, the PhenoAge clock, the SkinBlood clock, as well as an epigenetic estimate of telomere length (DNAmTL) using the online DNA Methylation Age Calculator (https://dnamage.genetics.ucla.edu/).

As expected, chronological age was strongly positively correlated with all epigenetic age estimators (Pearson r ranging from 0.79 to 0.93, Supplementary Figure 1), and these aging clocks were also strongly correlated with each other (Pearson r ranging from 0.73 to 0.90, Supplementary Figure 1). Meanwhile, the epigenetic estimate of telomere length, DNAmTL, was negatively correlated with chronological age and the epigenetic age estimates (Pearson r ranging from -0.63 to -0.72, Supplementary Figure 1).

**Figure 1:**
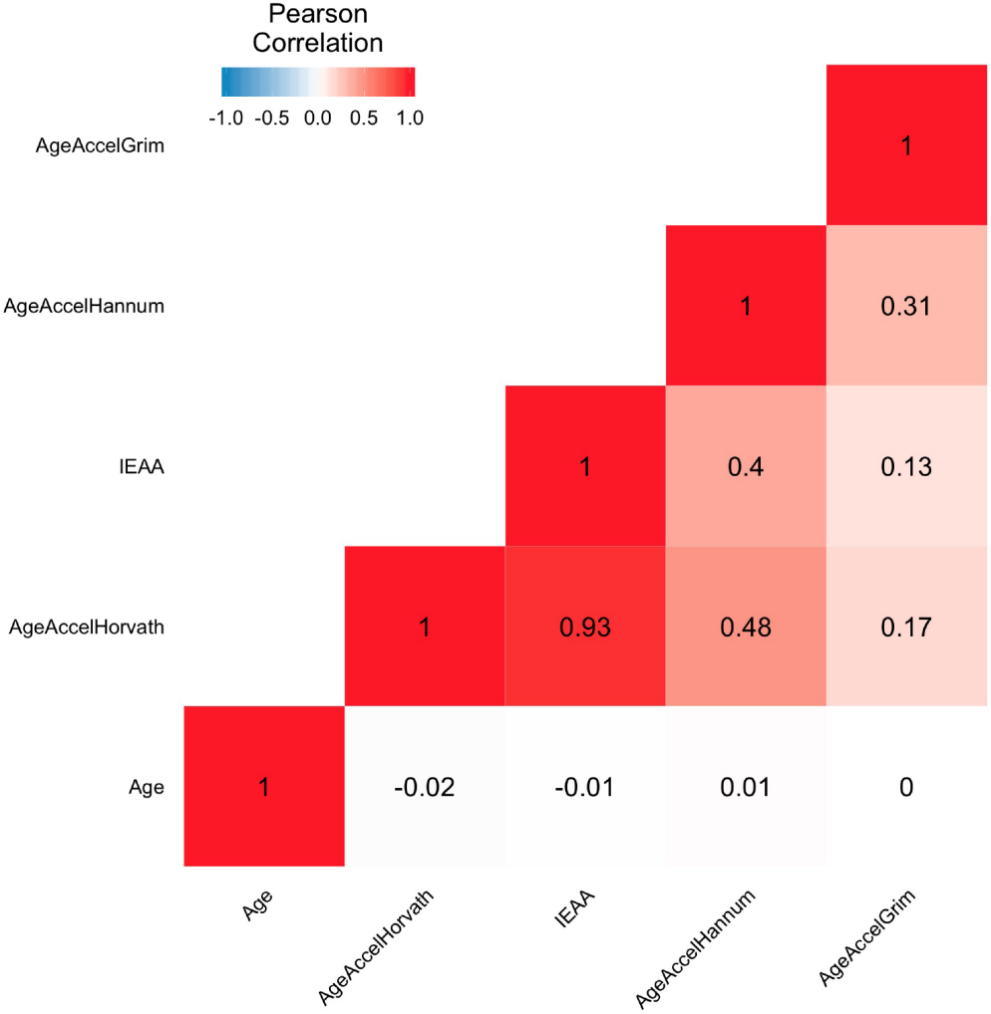
**Heatmap of pairwise correlations of chronological age and epigenetic age accelerations.** The heat map color-codes the pairwise Pearson correlations of chronological age and epigenetic age accelerations in the Framingham Heart Study (N=2326). Age represents the chronological age. AgeAccelHorvath, IEAA, AgeAccelHannum, and AgeAccelGrim represent measures of epigenetic age acceleration derived from the Horvath pan tissue clock, the intrinsic clock, the Hannum clock, and the GrimAge clock, respectively. The shades of color (blue, white, and red) visualize correlation values from -1 to 1. Each square reports a Pearson correlation coefficient.

For each clock, we calculated DNA methylation-based age acceleration based on the residuals of the regression of DNA methylation age on each participants’ chronological age. Thus, due to this approach, none of the epigenetic measures of age accelerations are correlated with chronological age (Pearson r = 0) as can be seen from Figure 1 and Supplementary Figure 2. AgeAccelHorvath is highly correlated (Pearson r = 0.93) with IEAA because both are based on the Horvath pan tissue clock. AgeAccelHannum was moderately associated with both AgeAccelHorvath and IEAA (Pearson r = 0.48 and 0.4 respectively). AgeAccelGrim showed only weak correlations with the other epigenetic measures of age acceleration which reflects the fact that GrimAge clock is a mortality risk predictor as opposed to an age estimator. Overall, the moderate pairwise correlations between the DNAm based biomarkers reflect different properties: some are highly confounded by blood cell composition and capture immunosenescence (Hannum, GrimAge, DNAmTL) while others are not (Horvath pan tissue, IEAA) [13, 32].

### Association between EML and DNAm aging clocks

**Figure 2:**
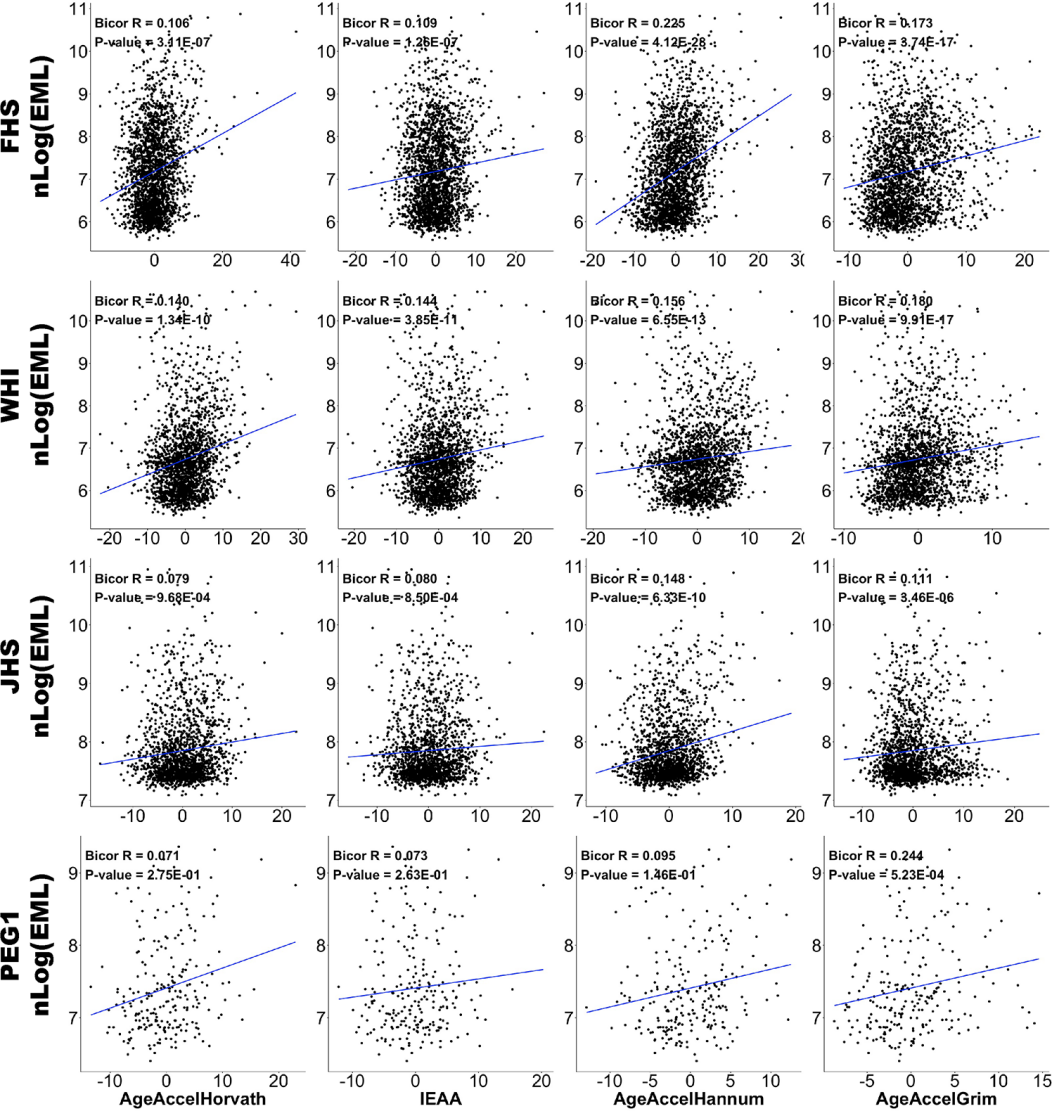
**Correlations between EML and epigenetic age accelerations.** Scatter plots of DNAm age acceleration estimators (x-axis; AgeAccelHorvath, IEAA, AgeAccelHannum, and AgeAccelGrim in each column, respectively) versus natural log-transformed EMLs (y-axis). Data from FHS, WHI, JHS, and PEG1 are plotted in four rows respectively. Each panel reports a biweight midcorrelation coefficient and correlation test p-value.

We estimated the association between EML, chronological age, cell composition, and DNAm age acceleration using biweight midcorrelation (bicor) for each dataset separately and calculated pooled statistics using Stouffer’s method. Bicor is a median-based measurement of correlation that is robust to outliers [33]. We adjusted for potential confounders including age, sex, race/ethnicity, and cell compositions (naïve CD8 cells, CD8+CD28-CD45RA- T cells, Plasma Blasts, CD4 T cells, and Granulocytes) by regressing out the effects of these factors and retaining the residuals only for analysis. Results for AgeAccelHorvath, IEAA, AgeAccelHannum, and AgeAccelGrim are shown in Table 2 and Figure 2, while other clocks can be found in Supplementary Table 2. These analyses show that EML per study participant was positively correlated with chronological age (meta r = 0.171, meta P-value = 1.64E-42). Furthermore, EML was negatively correlated with CD4+ T cells (meta r = -0.121, meta P-value = 4.24E-22), plasmablasts (meta r = -0.085, meta P-value = 1.14E-11), and granulocytes (meta r = -0.064, meta P-value = 3.70E-07), but positively with exhausted CD8+ (defined as CD8+CD28-CD45RA-) T cells. These results are consistent with known age-related changes in blood cell composition [34, 35].

**Table 2:** Biweight midcorrelation analysis of EML.

Outcome = log(EML) ^**^	Meta ^*^		**FHS (n = 2326)**		**WHI (n= 2091)**		**JHS (n= 1734)**		**PEG 1 (n = 237)**	
	**Meta r**	**Meta P_value**	**Bicor r**	**P_value**	**Bicor r**	**P_value**	**Bicor r**	**P_value**	**Bicor r**	**P_value**
Age	0.171	1.64E-42	0.244	7.15E-33	0.104	1.73E-06	0.145	1.50E-09	0.176	6.45E-03
DNAm Age Acceleration										
AgeAccelHorvath	0.109	3.25E-18	0.106	3.11E-07	0.140	1.34E-10	0.079	9.68E-04	0.071	2.75E-01
IEAA	0.112	4.04E-19	0.109	1.26E-07	0.144	3.85E-11	0.080	8.50E-04	0.073	2.63E-01
AgeAccelHannum	0.179	2.43E-46	0.225	4.12E-28	0.156	6.55E-13	0.148	6.33E-10	0.095	1.46E-01
AgeAccelGrim	0.162	2.25E-38	0.173	3.74E-17	0.180	9.91E-17	0.111	3.46E-06	0.224	5.23E-04
Cell types										
CD8.naive	-0.021	9.19E-02	-0.072	5.20E-04	0.020	3.66E-01	-0.011	6.58E-01	0.042	5.23E-01
CD8pCD28nCD45RAn	0.077	9.23E-10	0.085	3.90E-05	0.086	8.16E-05	0.052	2.88E-02	0.082	2.07E-01
PlasmaBlast	-0.085	1.14E-11	-0.054	8.94E-03	-0.070	1.39E-03	-0.140	4.89E-09	-0.110	9.23E-02
CD4T	-0.121	4.24E-22	-0.146	1.68E-12	-0.113	2.17E-07	-0.096	6.79E-05	-0.118	6.95E-02
Gran	-0.064	3.70E-07	-0.075	2.72E-04	-0.016	4.74E-01	-0.091	1.60E-04	-0.170	8.67E-03

^*^Meta-analysis using Stouffer’s method with weights given by the square root of the number of (non-missing) samples in each data set.

^**^Adjusted for Age, Sex, Race/ethnicity, Cell types.

EML was also positively correlated with AgeAccelHorvath, IEAA, AgeAccelHannum, and AgeAccelGrim, with AgeAccelHannum exhibiting the strongest correlation (meta r = 0.179; meta P-value = 2.43E-46).

We further distinguished between epigenetic age acceleration and deceleration to determine correlations with EML. The correlation between EML and age acceleration was largely the same as what we presented originally. Interestingly, the correlation between EML and age deceleration was much smaller in size and less statistically significant (see Supplementary Table 3).

### Sensitivity analyses

We evaluated associations between EML, chronological age, cell compositions, and age accelerations in males and females separately (Supplementary Table 4). For both sexes, EML remained positively correlated with chronological age, exhausted CD8+ T cells, and age acceleration suggesting that EML and age acceleration are independent of sex.

Several sensitivity analyses were conducted to ensure the reliability and reproducibility of the observed associations. To address a possibly non-linear relationship between epigenetic aging and chronological age, we additionally adjusted for a square term in age, (age^2^, Supplementary Table 5). Also, to assess the potential for additional confounding, we adjusted for body mass index (Supplementary Table 6). This led to qualitatively similar results.

In order to explore whether the criteria used to define SEM will change results, we conducted another sensitivity analysis using two new SEM measures: 1) loose SEM: defined as a specific CpG site with its methylation level exceeding two times the interquartile range (IQR) of the first quartile (Q1 – 2 × IQR) or the third quartile (Q3 + 2 × IQR) across all subjects; and 2) stringent SEM: defined as a specific CpG site with its methylation level exceeding four times the interquartile range (IQR) of the first quartile (Q1 – 4 × IQR) or the third quartile (Q3 + 4 × IQR) across all subjects. We then calculated the total number of SEMs according to the loose and stringent definition for each person (loose or stringent EML, respectively). The biweight midcorrelations between loose or stringent EMLs and measures of epigenetic age accelerations were very similar to the original results (Supplementary Table 7).

We also explored the effect of different normalization methods for the methylation data (Illumina background correction, functional normalization, Noob, and quantile normalization). We found that the association between EML and age acceleration was not influenced by the normalization method (Supplementary Table 8).

### Functional annotations

To test whether individual SEMs were randomly distributed across the genome or were more likely to be found in certain genomic regions or biological pathways, we conducted enrichment analyses to assess whether SEMs were enriched in clock-CpGs (Horvath clock, PhenoAge clock, Hannum clock), genomic regions (transcription start sites (TSS1500, TSS200), untranslated regions (5’UTR, 3’UTR), 1^st^ Exon, and gene body), or regulatory features (i.e., enhancers, DNase hypersensitive sites, open chromatin regions, transcription factor binding site, promoters). For each participant, we first annotated the probes and each mutation based on the location related to genes (i.e., TSS1500, TSS200, 5’UTR, 1^st^ EXON, gene body, 3’UTR), or regulatory features using the manifest provided by Illumina. Then we conducted hypergeometric tests for each region and each subject separately using a nominal significance threshold of 0.05. Last, for each region, we summarized the number of individuals for which the test was significant (Supplementary Tables 9–11).

The results show that for each clock-CpG set or region, only a small proportion of participants have their SEMs enriched which illustrating both the stochastic nature and inter-personal variation of SEMs.

### Pathway enrichment analysis

Next, we examined whether, among study participants who exhibit faster age accelerations, SEMs are enriched in particular biological pathways. We first conducted KEGG pathway enrichment analyses for each study participant. Then for each KEGG pathway, we calculated the number of people with enriched SEMs for this particular pathway. Finally, we investigated the association between SEMs enrichment in a particular pathway and age accelerations using linear regression. Additional details can be found in the method section.

Supplementary Tables 12–16 showed the top 10 pathways that were commonly enriched in each study, and generally we found that SEMs enriched in these pathways were also statistically significantly associated with faster age accelerations (AgeAccelHorvath, IEAA, AgeAccelHannum, and AgeAccelGrim, respectively).

Briefly, in all four study populations, we identified similar enrichment patterns, and SEMs enriched in signaling pathways, axon guidance, glutamatergic synapse, morphine addiction, glucocorticoid pathway (Cushing syndrome), or circadian rhythm pathways were associated with faster AgeAccelHorvath, AgeAccelHannum, and IEAA. Whereas the associations between pathway enrichment and AgeAccelGrim or AgeAccelPheno were less strong and not necessarily statistically significant. We only observed SEMs enriched in neuroactive ligand-receptor interaction was associated with faster AceAccelGrim and AgeAccelPheno.

### Region-specific EML

To address the functionality of SEMs on biological age acceleration, we calculated the number of SEMs co-located with clock CpGs for each study participant (i.e., clock-specific SEMs) and assessed whether there were any clock-specific EMLs corresponding to age acceleration (Supplementary Table 17), but we observed no statistically significant association with the three clocks tested (Horvath clock: 353 CpGs; Hannum clock: 71 CpGs; PhenoAge clocks: 513 CpGs).

Next, we divided CpGs into different genomics region/regulatory feature groups based on the annotations, and then calculated EMLs within each region for each study participant (i.e., genomic region-specific EML; regulatory region-specific EML) (Table 3 and Supplementary Table 18). EMLs in TSS1500, TSS200, and the 1stExon regions were related to faster age accelerations. Also, EML in DNase hypersensitive regions was positively correlated with faster age accelerations. In contrast, 3’UTR specific-EML was associated with younger chronologic age and slower age acceleration.

**Table 3:** Meta-analysis ^*^: Biweight midcorrelation analysis of genomic region-specific EML.

Outcome = log(Region-EML) ^**^	**TSS1500 (50999 CpGs)**		**TSS200 (41175 CpGs)**		**5'UTR (23024 CpGs)**		**1stExon (7669 CpGs)**		**Gene Body (135960 CpGs)**		**3'UTR (14010 CpGs)**	
	**Meta r**	**Meta P_value**	**Meta r**	**Meta P_value**	**Meta r**	**Meta P_value**	**Meta r**	**Meta P_value**	**Meta r**	**Meta P_value**	**Meta r**	**Meta P_value**
Age	0.103	1.42E-16	0.074	3.39E-09	-0.046	2.44E-04	0.126	7.86E-24	-0.122	1.81E-22	-0.085	1.38E-11
DNAm Age Acceleration												
AgeAccelHorvath	0.050	7.35E-05	-0.004	7.52E-01	-0.055	9.43E-06	0.074	4.13E-09	-0.060	1.96E-06	-0.064	3.35E-07
IEAA	0.058	3.16E-06	-0.006	6.52E-01	-0.052	2.94E-05	0.067	9.83E-08	-0.063	4.04E-07	-0.064	2.66E-07
AgeAccelHannum	0.098	5.06E-15	0.076	1.62E-09	-0.044	4.93E-04	0.154	1.00E-34	-0.140	4.87E-29	-0.111	6.74E-19
AgeAccelGrim	-0.001	9.40E-01	0.054	1.45E-05	-0.012	3.57E-01	0.082	6.30E-11	-0.053	1.94E-05	-0.073	5.52E-09
Cell types												
CD8.naive	-0.048	1.23E-04	-0.035	5.18E-03	-0.023	6.55E-02	-0.044	4.66E-04	0.053	1.96E-05	0.037	3.10E-03
CD8pCD28nCD45RAn	0.043	5.51E-04	-0.032	1.07E-02	0.015	2.25E-01	-0.020	1.08E-01	0.016	2.06E-01	-0.018	1.41E-01
PlasmaBlast	0.041	1.04E-03	-0.019	1.28E-01	-0.019	1.23E-01	-0.022	7.45E-02	-0.023	6.96E-02	-0.012	3.32E-01
CD4T	0.043	5.19E-04	-0.066	1.34E-07	-0.013	2.91E-01	-0.095	2.97E-14	0.002	8.74E-01	0.024	5.09E-02
Gran	-0.008	5.43E-01	-0.082	5.17E-11	-0.039	1.85E-03	-0.113	1.82E-19	0.067	9.65E-08	0.065	1.72E-07

^*^ Meta-analysis using Stouffer’s method with weights given by the square root of the number of (non-missing) samples in each data set.

^**^ Adjusted for Age, Sex, Race/ethnicity, Cell types, Log(total EML).

### The direction of SEM

Based on the direction of the mutation, we separated SEMs into hypomethylated SEM (Q1 – 3 × IQR) and hypermethylated SEM (Q3 + 3 × IQR) and calculated hypomethylated and hypermethylated EMLs respectively within FHS. We assessed the correlations between the newly calculated directional EMLs and epigenetic age acceleration. The results remained largely the same (See Supplementary Table 19). Furthermore, consistent with previous studies [29], hypermethylated SEMs were mainly located in CpG islands, while hypomethylated SEMs were enriched in the open seas (see Supplementary Tables 20, 21).

### Shannon entropy, EML, and DNAm age acceleration

As an alternate measure for a well-functioning EMS that maintains genomic stability, we calculated the Shannon entropy of the whole methylome based on the 450K or EPIC array. An increase in entropy means that the methylome becomes less predictable across the population of cells, i.e. when the methylation fractions (beta values) tend towards 50%.

We found the Shannon entropy to be positively correlated with chronologic age (meta r = 0.046, meta P-value = 2.16E-04), EML (meta r = 0.234, meta P-value = 7.71E-78), and all four measures of age accelerations (meta P-value: AgeAccelHorvath = 8.79E-09, IEAA = 6.56E-04, AgeAccelHannum = 1.80E-22, AgeAccelGrim = 1.67E-22) (Table 4 and Supplementary Table 22).

**Table 4:** Association between Shannon entropy and age, AgeAccel, EML.

Outcome =Entropy ^**^	Meta ^*^		**FHS (n = 2326)**		**WHI (n= 2091)**		**JHS (n= 1734)**		**PEG 1 (n = 237)**	
	**Meta r**	**Meta P_value**	**Bicor r**	**P_value**	**Bicor r**	**P_value**	**Bicor r**	**P_value**	**Bicor r**	**P_value**
Age	0.046	2.16E-04	0.001	9.55E-01	0.068	2.01E-03	0.071	2.92E-03	0.117	7.30E-02
DNAm Age Acceleration										
AgeAccelHorvath	0.072	8.79E-09	0.081	9.11E-05	0.160	1.76E-13	-0.039	1.02E-01	0.006	9.22E-01
IEAA	0.043	6.56E-04	0.035	9.02E-02	0.131	1.70E-09	-0.052	3.16E-02	0.018	7.83E-01
AgeAccelHannum	0.122	1.80E-22	0.155	6.23E-14	0.136	4.60E-10	0.063	9.10E-03	0.096	1.41E-01
AgeAccelGrim	0.122	1.67E-22	0.077	1.89E-04	0.228	3.86E-26	0.043	7.17E-02	0.164	1.14E-02
EML	0.234	7.71E-78	0.089	1.63E-05	0.294	6.87E-43	0.325	7.22E-44	0.281	1.10E-05

^*^Meta-analysis using Stouffer’s method with weights given by the square root of the number of (non-missing) samples in each data set.

^**^Adjusted for Age, Sex, Race/ethnicity, Cell types.

## DISCUSSION

It has previously been proposed that aging-related decline in epigenetic maintenance increases the occurrence of SEMs in individuals [24, 25]. Our data suggest that the EML per study participant are weakly but statistically significantly associated with several widely used measures of epigenetic age acceleration based on epigenetic clocks.

It has been hypothesized that DNA methylation clocks may capture the imperfection of the EMS resulting in epigenetic instability [5, 13, 21]. Our study provides new evidence for this hypothesis showing that the accumulation of stochastic epigenetic mutations is associated with epigenetic age acceleration according to four clocks: the Horvath, the intrinsic, the Hannum, and the GrimAge clock. The first three clocks have been built to predict chronological age while the GrimAge clock was designed as a mortality risk predictor that explicitly uses chronological age as one of its predictors. We observed statistically significant associations between EML and age acceleration measured by all four clocks, with the AgeAccelHannum and AgeAccelGrim being most strongly associated with EML. One explanation might be that these clocks have different relationships to blood cell composition. While measures of epigenetic age acceleration based on Horvath's pan tissue clock, AgeAccelHorvath and IEAA, are at best weakly related to changes in blood cell composition, AgeAccelHannum and AgeAccelGrim correlate more strongly with blood cell counts and markers of immunosenescence. Therefore, similar to the Hannum and GrimAge clocks, EML also reflects changes in blood cell composition i.e. the immune system. Previously, studies showed that DNAm biomarkers of aging that capture altered immune cell composition are better predictors of mortality [7, 13]. Thus, not only the intracellular accumulation of epigenetic mutations we investigated here, but also changes in cell composition contribute to EML as part of the biological aging processes that diverge from chronological age. This finding is also consistent with several previous studies [25, 27, 29].

It is worth noting that only the intracellular accumulation of epigenetic mutations suggests that an insufficient EMS may be involved in increasing the EML, thus we ascribe greater weight and importance to the correlations with the Horvath clock and IEAA. The relatively weak correlations between EML and AgeAccelHorvath or IEAA indicate that these DNAm age estimators also capture other hallmarks of the aging process apart from a dysfunctional EMS [3]. The size of the correlations for these age acceleration measures and EML are comparable to those reported for many other known risk factors of aging. For example, in the WHI cohort, the correlation between BMI and AgeAccelGrim is 0.14; the correlation between exercise and AgeAccelGrim is -0.1; and the correlations for many other risk factors are below +/- 0.3 (See Lu et al) [7]. Correlations between risk factors and IEAA are even smaller (r between 0.08 and -0.06, see Quach et al. Figure 1) [36]. Nevertheless, the fact that associations between EML and AgeAccelHorvath or IEAA are, at best, weak reminds us that other mechanisms and factors apart from EMS also play important roles in the ageing process. Indeed, future in vivo or in vitro studies are needed to better understand the causal relationship between EML and epigenetic aging.

EML exhibited much stronger correlations with age acceleration than deceleration. This result suggests that epigenetic age acceleration and deceleration may have different biological mechanisms, and that the maintenance of epigenetic stability plays more of a role in the acceleration of epigenetic age than the deceleration.

Our finding that EML is statistically significantly albeit weakly correlated with various measures of epigenetic age acceleration is consistent with several previous studies [27, 29]. In order to understand the biological foundation for EML contributions to the epigenetic aging process, we conducted several functional and pathway enrichment analyses. Functional annotation and pathway enrichment analysis showed no predominant regions or biological pathways as being enriched with SEMs. This is in line with previous observations that a majority of SEMs are randomly distributed across the genome and that the locations necessarily differ between individuals as the name suggests [27]. Despite this inherent inter-individual variation, we found that individuals with SEMs enriched in signaling pathways, neurogenesis, neurotransmission, glucocorticoid, or circadian rhythm pathways were more likely to show age acceleration as measured by AgeAccelHorvath, IEAA, and AgeAccelHannum. These non-random patterns – if confirmed – may very well reflect the accumulation of SEMs in pathways related to biological mechanisms that are involved in aging. For example, some signaling pathways such as oxytocin signaling and MAPK/ERK signaling pathway have been associated with age-related muscle maintenance and regeneration [37], while excess glucocorticoid levels may reflect a lifelong accumulation of stressors and this pathway plays a key role in frailty [38] and the aging process [21]. Furthermore, some clock-CpGs are located in glucocorticoid response elements [39]. We also found SEMs enriched in several neurogenesis or neurotransmission-related pathways that may be contributing to the ticking rate of clocks. This is consistent with the previous finding that DNAm age acceleration is linked to neuropathology [18, 40], especially Parkinson’s disease [16] and Down syndrome [15]. Moreover, our finding of SEM enrichment in the circadian entrainment pathway supports the hypothesis that the DNAm age estimators are related to the oscillation of the circadian rhythm [13]. Interestingly, although patterns were similar, we found less evidence for pathway enrichment with SEMs and age acceleration based on the GrimAge and PhenoAge clocks. This may again underscore that different clocks indeed capture different aspects of the aging process.

EMLs within TSS1500, TSS200, and especially the 1stExon regions were found to be associated with faster age accelerations, and for these regions methylation levels have been shown to be related to gene expression [41, 42]. Therefore, our result may suggest that the accumulation of random epigenetic mutations in these regions may influence biological aging processes through gene expression regulation. Interestingly, even though we would also have expected this, we did not observe such associations for promoter regions. Further studies are needed to investigate the biologic consequences of region-specific effects of epigenetic mutations on aging.

The Shannon Entropy measure reflects higher levels of entropy such that the methylome becomes less predictable across the population of cells due to the failure of DNAm maintenance [11]. Epigenetic Shannon entropy as well as this measure’s variability increase with age [10, 11, 43, 44]. In our study, EML and Shannon entropy was strongly correlated, confirming that both measure aspects of the EMS, even though EML and entropy capture different aspects of epigenetic stability. SEM represents rare methylation value extremes at a site due to the accumulation of maintenance failures whereas entropy reflects an ongoing ‘smoothing’ of the epigenetic landscape such that beta values tend towards 50% [45].

There are limitations of our study. First, it is possible that some unmeasured confounders biased our results. Sensitivity analyses, however, showed that the SEM measure was not affected by potential technical artifacts or poor sample quality, and the association between EML and age acceleration was independent of potential confounders including chronological age, sex, race/ethnicity, and BMI. Hence, although technical effects and confounding are hard to avoid, the observed associations between EML and age accelerations were robust to adjustments for a number of covariates. Second, from all four studies, we only had cross-sectional data available. Therefore, we were unable to investigate the accumulation of epigenetic mutations over time within individuals. Finally, DNA methylation was measured in blood samples only. Therefore, pathway results need to be interpreted with caution as many of the identified pathways listed above have no direct relevance to the function of the blood tissue. While this seems to support the inherent randomness of SEMs, stochastic epigenetic mutations may still accumulate in a non-random pattern within certain biological pathways if repair mechanisms fail systematically due to properties related to these pathways across different tissues. Also, it has been shown that epigenetic changes in blood may indeed reflect epigenetic fingerprints of other target tissues [46, 47]. Nevertheless, tissue- and cell-specific analyses are needed to better understand the relationship between stochastic epigenetic mutations and aging processes in different tissues.

In summary, using large datasets from multiple population-based studies, we were able to show that EML per study participant is associated with different epigenetic aging markers (aging clocks) and importantly with epigenetic age acceleration. Moreover, epigenetic mutations enriched in particular biological pathways or genomic regions related to gene expression were associated with accelerated aging and these may contribute to the ticking of the epigenetic clock. Our findings from pathway enrichment analyses also suggest some interesting biological mechanisms that may influence the ticking of the epigenetic aging clocks and drive the acceleration of the biological aging process.

## MATERIALS AND METHODS

### Study population

Our study is based on data from four studies: the Framingham Heart Study (FHS) Offspring Cohort, the Women’s Health Initiative (WHI), the Jackson Heart Study (JHS), and the Parkinson’s Environment and Genes (wave 1) known as the PEG1 study.

We used 2,326 individuals from the FHS Offspring cohort [48]. The FHS cohort is a large-scale longitudinal study started in 1948, initially investigating the common factors of characteristics that contribute to cardiovascular disease (CVD) (https://www.framinghamheartstudy.org/index.php). The study at first enrolled participants living in the town of Framingham, Massachusetts, who were free of overt symptoms of CVD, heart attack, or stroke at enrollment. In 1971, the study started FHS Offspring Cohort to enroll a second generation of the original participants’ adult children and their spouses (n= 5124) for conducting similar examinations. The FHS Offspring Cohort collected medical history and measurement data, immunoassays at exam 7, and blood DNA methylation profiling at exam 8. Participants from the FHS Offspring Cohort were eligible for our study if they attended both the seventh and eighth examination cycles and consented to have their molecular data used for the study. We used the 2,326 participants from the group of Health/Medical/Biomedical (IRB, MDS) consent and available for both Immunoassay array DNA methylation array data. The FHS data are available in dbGaP (accession number: phs000363.v16.p10 and phs000724.v2.p9).

The WHI is a national study that enrolled postmenopausal women aged 50-79 years into the clinical trials (CT) or observational study (OS) cohorts between 1993 and 1998 [49, 50]. We included 2,091 WHI participants with available phenotype and DNA methylation array data from “*Broad Agency Award 23*” (WHI BA23). WHI BA23 focuses on identifying miRNA and genomic biomarkers of coronary heart disease (CHD), integrating the biomarkers into diagnostic and prognostic predictors of CHD and other related phenotypes, and other objectives can be found in https://www.whi.org/researchers/data/WHIStudies/StudySites/BA23/Pages/home.aspx.

The JHS is a large, population-based observational study evaluating the etiology of cardiovascular, renal, and respiratory diseases among African Americans residing in the three counties (Hinds, Madison, and Rankin) that make up the Jackson, Mississippi metropolitan area [51]. The age at enrollment for the unrelated cohort was 35-84 years; the family cohort included related individuals >21 years old. Participants provided extensive medical and social history, had an array of physical and biochemical measurements and diagnostic procedures, and provided genomic DNA during a baseline examination (2000-2004) and two follow-up examinations (2005-2008 and 2009-2012). Annual follow-up interviews and cohort surveillance are ongoing. In our analysis, we used the visits at baseline from 1,734 individuals as part of project JHS ancillary study ASN0104, available with both phenotype and DNA methylation array data.

The PEG1 study was conducted during 2000-2007 to investigate the causes of Parkinson's disease (PD) in agricultural regions of the California central valley. We analyzed blood samples from 238 healthy controls enrolled from Kern, Tulare, or Fresno counties. Controls were required to be over the age of 35, having lived within one of the counties for at least 5 years prior to enrollment, and do not have a diagnosis of Parkinsonism. Demographic information, lifestyle factors, and medication use were collected in standardized interviews, including lifetime information of cigarette smoking and coffee/ tea consumption.

For PEG1, FHS, and WHI, peripheral blood samples were collected, and bisulfite conversion using the Zymo EZ DNA Methylation Kit (Zymo Research, Orange, CA, USA) as well as subsequent hybridization of the HumanMethylation450k Bead Chip (Illumina, San Diego, CA), and scanning (iScan, Illumina) were performed according to the manufacturer’s protocols by applying standard settings. For JHS, DNA methylation quantification was conducted using HumanMethylation EPIC Bead Chip (Illumina, San Diego, CA).

### Preprocess

For PEG1 samples, raw signal intensities were retrieved using the function *read.metharray.exp* of the R package *minfi* from the Bioconductor open-source software (http://www.bioconductor.org/), followed by linear dye bias correction, noob background correction, and functional normalization using the same R package [52–55]; β-value was used for all the analyses. One sample was identified as low quality due to low median methylated and unmethylated signal intensities across the entire array and thus removed from the study population. Detection p-values were derived using the function *detectionP* as the probability of the total signal (methylation + unmethylated) being detected above the background signal level, as estimated from negative-control probes. All in all, 845 probes with a detection p-value above 0.05 in at least 5% of samples were removed. Also, 645 probes with a bead count <3 in at least 5% of samples; 11,334 probes on the X or Y chromosome; 7,306 probes containing a SNP at the CpG interrogation site and/or at the single nucleotide extension for 5% maf; and 27,332 cross-reactive probes were also removed. In total, 438,050 probes were included for downstream analyses.

For FHS and WHI samples, 11,334 probes on the X or Y chromosome; 7,306 probes containing a SNP at the CpG interrogation; and 27,332 cross-reactive probes were also removed. In total, 439,540 probes were included for downstream analyses.

For JHS samples, 19,532 probes on the X or Y chromosome; 53,435 probes containing a SNP at the CpG interrogation and cross-reactive were also removed. In total, 793,869 probes were included for downstream analyses.

### SEM calculation

The calculation of SEM was consistent with a previously published and validated approach [24, 26, 31]. CpG with methylation levels three times the interquartile range above the third quartile or below the first quartile was identified as a SEM. Toward this end, we calculated the IQR for each of the 438,050 probes in each dataset (for PEG1, FHS, and WHI) or the 793,969 probes (for JHS). Then, SEMs were identified based on extreme methylation levels. Finally, we summed across the count of all SEMs per sample and defined the total number of SEMs of each study participant as epigenetic mutation load (EML). EML was not normally distributed; therefore, we used the natural log of the number of SEMs for all regression analyses.

In FHS, we separated SEMs into hypermethylated and hypomethylated SEMs based on the direction of the mutation. We also defined consistently hypermethylated or hypomethylated SEMs as a CpG mutated in the same direction in more than 10 participants.

In order to assess whether the criteria used for SEMs will change the results, we defined loose SEM and stringent SEM as described above. We then calculated the total numbers of loose and stringent SEMs for each person.

### DNA methylation age

We included eight different DNAm aging biomarkers in this study. Utilizing our online DNA Methylation Age Calculator (https://dnamage.genetics.ucla.edu/), we calculated DNA methylation-based ages and the age accelerations based on the residuals of the regression of DNA methylation age on each participants’ chronological age for each clock.

Four types of DNA methylation-based biomarkers were included in the main analyses. Briefly, Horvath clock was calculated using a linear combination of 353 CpGs that have previously been shown to predict chronological age in multiple tissues [5]; and the intrinsic clock was derived from the Horvath clock by additionally regressing out cell compositions [30]; Hannum clock was calculated using a linear combination of 71 CpGs to predict chronological age in blood [11]; and GrimAge clock) was calculated from a linear combination of 7 DNAm plasma protein surrogates and a DNAm-based estimator of smoking pack-years designed to predict mortality [7].

Other DNAm aging biomarkers were included in the Supplementary analyses, including: the extrinsic clock [30], PhenoAge clock [6], SkinBlood clock [56], DNAm based estimator of telomere length [57], each of the 7 DNAm protein surrogates underlying the definition of the GrimAge clock [7], as well as DNAm based estimate of smoking pack-years.

### Cell composition

White blood cell composition was imputed for each study participant using our online published DNA Methylation Age Calculator, https://dnamage.genetics.ucla.edu/. The following imputed blood cell counts were included in downstream analyses: CD4+ T, naïve CD8+ T, exhausted cytotoxic CD8+ T cells (defined as CD8 positive CD28 negative CD45R negative, CD8+CD28-CD45RA-), plasmablasts, and granulocytes. Naïve CD8+ T, exhausted cytotoxic CD8+ T cells, and plasmablasts were calculated based on the Horvath method [58]. The remaining cell types were imputed using the Houseman method [59].

### Shannon entropy

The formula of Shannon entropy is:

$$\begin{array}{l} Entropy=\frac{1}{N\;\times \;\;log_{2}(\frac{1}{2})}\\ \;\;\;\;\;\;\;\;\;\;\;\;\;\;\;\;\;\;\;\;\;\;\;\;\;\;\;\;\;\times \;\sum_{i=1}^{N}[\beta_{i}\times \;log_{2}(\beta_{i})+(1-\beta_{i})\\ \;\;\;\;\;\;\;\;\;\;\;\;\;\;\;\;\;\;\;\;\;\;\;\;\;\;\;\;\times \;\;log_{2}(1-\beta_{i})] \end{array}$$

where *β_i_* represents the methylation beta value for the i^th^ probe (CpG site) in the array, N represents the total number of probes included in the formula [11].

### Statistical analysis

All analyses were conducted using R v3.6.1. We used Pearson correlations to assess the relations between different DNAm ages and age accelerations. We evaluated potential batch effects by assessing the difference of EMLs in microarray slides or position on the array with the ANOVA test. To eliminate the possibility that SEMs are driven by incomplete bisulfite conversion, Pearson correlations between EMLs and the average intensity of bisulfite conversion controls were also calculated. Average intensity of bisulfite conversion controls was derived using the *ENmix* R package [60].

To assess the association between EML and age/age accelerations/cell compositions, we applied biweight midcorrelation (bicor) implemented in the WGCNA R package. We adjusted for potential confounders including age, sex, race/ethnicity, and cell compositions (naïve CD8 cells, CD8+CD28-CD45RA- T cells, Plasma Blasts, CD4 T cells, and Granulocytes) by regressing out the effects of these factors and retaining the residuals of log(EML)only for analysis.

We also conducted stratified analyses to evaluate the associations between EML, chronological age, epigenetic estimates of cell composition, and epigenetic age acceleration in males and females separately for the PEG1, FHS, and JHS studies. To ensure the reliability and reproducibility of the associations, several sensitivity analyses were conducted. In addition to the potential confounders mentioned above, we adjusted for a quadratic term in age (age^2) to account for non-linear relationships and body mass index.

To evaluate the effect of data preprocessing steps, we repeated the analysis using four different normalization methods (Illumina background correction, noob, functional normalization, and quantile normalization implemented in the *minfi* package) in the PEG dataset.

We analyzed each dataset separately, therefore in order to obtain an overall p-value across all four studies, we conducted meta-analyses using Stouffer’s method for meta-analysis estimates of the correlation coefficient (meta r), and the corresponding two-sided p-values (meta p-value).

### Functional annotation, region-specific SEMs, and pathway enrichment analysis

For each participant, we annotated the probes and SEMs based on the location in relation to genes (TSS1500, TSS200, 5’UTR, 1^st^ Exon, gene body, and 3’UTR), or regulatory features (enhancer region, DNase hypersensitive region, open chromatin region, transcription factor binding site, and promoter region) using the manifest provided by Illumina. To test for region specific enrichment, we conducted hypergeometric tests for each region and each subject separately. A p-value less than 0.05 was considered statistically significant. For biological pathway enrichment analysis, the *enrichKEGG* function implemented in the *ClusterProfiler* package [61] was used to assess whether study participant’s SEMs were enriched in particular KEGG pathways (P-value threshold = 0.05). For each genomic region or KEGG pathway, we then summarized how many study participants had their SEMs enriched. We also investigated the association between SEMs enrichment in each KEGG pathway and age accelerations using linear regression, adjusted for the total number of SEMs per study participant:

$$\begin{array}{l} For\;pathway\;j:AgeAccel_{i}\\ \;\;\;\;\;\;\;\;\;\;\;\;\;\;\;\;\;\;\;\;\;\;\;\;\;\;\;\;\;\;\;\;=\beta_{0}\;+\;\beta_{1}\;Enrich_{ij}\\ \;\;\;\;\;\;\;\;\;\;\;\;\;\;\;\;\;\;\;\;\;\;\;\;\;\;\;\;\;\;\;+\;\beta_{2}\;\log\;(Total\;EML_{i})\;+\;\varepsilon_{i} \end{array}$$

where *AgeAccel* stands for age accelerations (AgeAccelHorvath, IEAA, AgeAccelHannum, AgeAccelGrim); *Enrich* stands for enrichment for pathway j (significant: 1, non-significant: 0); *Total EML* stands for log transformed total EML for participant i.

## Supplementary Material

Supplementary Figures

Supplementary Tables
