# Supplementary material for: Epigenetic mutation load is weakly correlated with epigenetic age acceleration
Source: Aging (Albany NY). 2020 Sep 29;12(18):17863–94. doi: 10.18632/aging.103950 (PMC7585066; doi:10.18632/aging.103950)
Supplement: Supplementary Figures [file aging-12-103950-s001..pdf]

SUPPLEMENTARY FIGURES

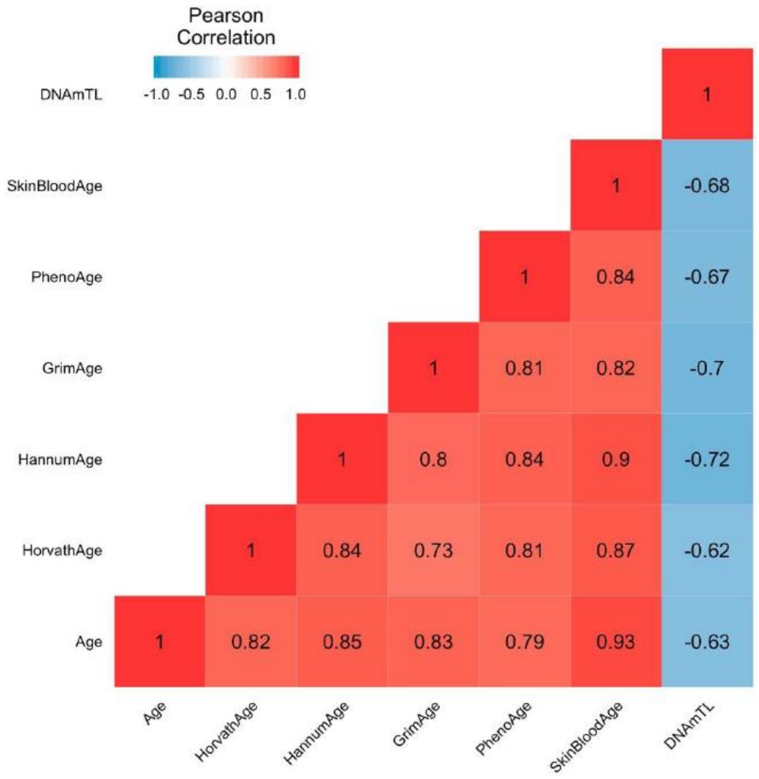

Supplementary Figure 1. Heatmap of pairwise correlations of chronological age and epigenetic ages. The heatmap color-codes the pairwise Pearson correlations of chronological age and epigenetic age in the Framingham Heart Study (N=2326). Age represents the chronological age. HorvathAge, HannumAge, GrimAge, PhenoAge, SkinBloodAge represent measures of epigenetic age derived from the Horvath pan tissue clock, the Hannum clock, the GrimAge clock, the PhenoAge clock, and the SkinBloodAge clock, respectively. DNAmTL represent DNAm-based surrogate markers of telomere length. The shades of color (blue, white, and red) visualize correlation values from -1 to 1. Each square reports a Pearson correlation coefficient.

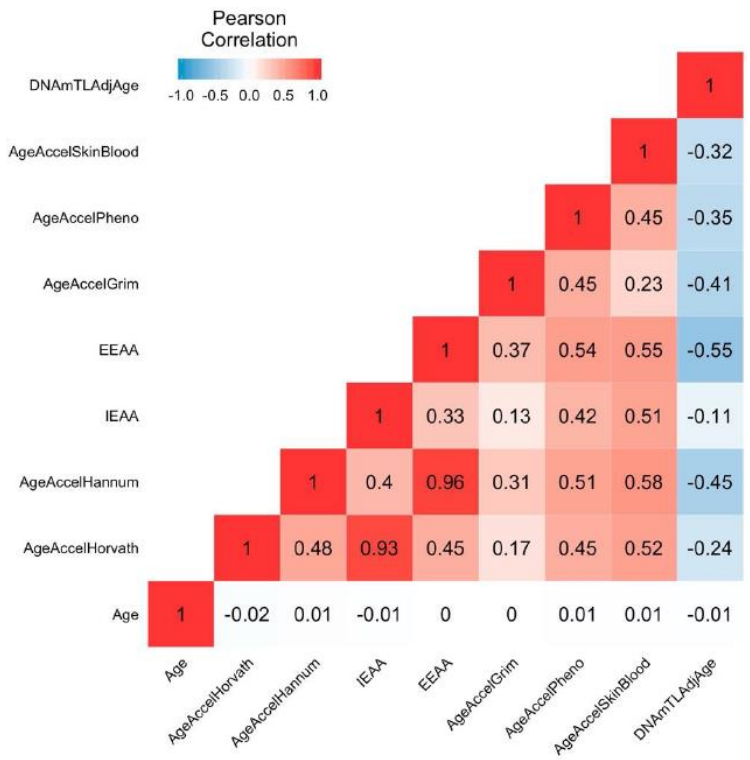

Supplementary Figure 2. Heatmap of pairwise correlations of chronological age and epigenetic age accelerations. The heatmap color-codes the pairwise Pearson correlations of chronological age and epigenetic age accelerations in the Framingham Heart Study (N=2326). Age represents the chronological age. AgeAccelHorvath, AgeAccelHannum, IEAA, EEAA, AgeAccelGrim, AgeAccelPheno, and AgeAccelSkinBlood represent measures of epigenetic age acceleration derived from the Horvath pan tissue clock, the Hannum clock, the intrinsic clock, the extrinsic clock, the GrimAge clock, the PhenoAge clock, the SkinBloodAge clock, respectively. DNAmTLAdjAge represents age adjusted DNAm-based surrogate markers of telomere length. The shades of color (blue, white, and red) visualize correlation values from -1 to 1. Each square reports a Pearson correlation coefficient.
