# Supplementary material for: Epigenetic mutation load is weakly correlated with epigenetic age acceleration
Source: Aging (Albany NY). 2020 Sep 29;12(18):17863–94. doi: 10.18632/aging.103950 (PMC7585066; doi:10.18632/aging.103950)
Supplement: Supplementary Tables [file aging-12-103950-s002..pdf]

SUPPLEMENTARY TABLES

Supplementary Table 1. Distribution of EML for each dataset.

	FHS (450K)	WHI (450K)	JHS (EPIC)	PEG1 (450K)
**min(EML)**	264	213	1194	603
**max(EML)**	52783	43768	56691	11645
**mean(EML)**	2433	1647	3401	2137
**sd(EML)**	4183	3937	4512	1953
**% of probes as SEM**	86%	85%	77%	45%

Supplementary Table 2. Biweight midcorrelation analysis of EML.

Outcome = log(EML) **	Meta *		FHS (n = 2326)		WHI (n= 2091)		JHS (n= 1734)		PEG 1 (n = 237)	
	Meta r	Meta P_value	Bicor r	P_value	Bicor r	P_value	Bicor r	P_value	Bicor r	P_value
Age	0.171	1.64E-42	0.244	7.15E-33	0.104	1.73E-06	0.145	1.50E-09	0.176	6.45E-03
BMI	0.005	7.94E-01	0.017	4.23E-01	-0.009	6.91E-01				
DNAm Age Acceleration										
AgeAccelHorvath	0.109	3.25E-18	0.106	3.11E-07	0.140	1.34E-10	0.079	9.68E-04	0.071	2.75E-01
IEAA	0.112	4.04E-19	0.109	1.26E-07	0.144	3.85E-11	0.080	8.50E-04	0.073	2.63E-01
EEAA (Unadjusted for cell types)	0.236	5.82E-79	0.297	1.12E-48	0.212	1.31E-22	0.166	3.09E-12	0.211	1.05E-03
AgeAccelHannum	0.179	2.43E-46	0.225	4.12E-28	0.156	6.55E-13	0.148	6.33E-10	0.095	1.46E-01
AgeAccelGrim	0.162	2.25E-38	0.173	3.74E-17	0.180	9.91E-17	0.111	3.46E-06	0.224	5.23E-04
DNAmADMAdjAge	0.068	4.75E-08	0.121	4.58E-09	0.070	1.41E-03	-0.017	4.71E-01	0.157	1.58E-02
DNAmB2MAdjAge	0.143	3.47E-30	0.189	4.69E-20	0.129	3.44E-09	0.074	2.05E-03	0.287	7.20E-06
DNAmCystatinCAdjAge	0.153	3.02E-34	0.119	8.71E-09	0.241	6.48E-29	0.081	7.30E-04	0.183	4.64E-03
DNAmGDF15AdjAge	0.148	3.64E-32	0.167	6.09E-16	0.127	5.80E-09	0.142	3.01E-09	0.162	1.27E-02
DNAmLeptinAdjAge	-0.025	4.65E-02	-0.021	3.19E-01	-0.035	1.11E-01	-0.031	2.03E-01	0.063	3.34E-01
DNAmPACKYRSAdjAge	0.161	8.96E-38	0.166	7.99E-16	0.172	2.23E-15	0.137	1.10E-08	0.150	2.13E-02
DNAmPAI1AdjAge	0.031	1.47E-02	0.024	2.49E-01	-0.004	8.59E-01	0.061	1.07E-02	0.173	7.56E-03
DNAmTIMP1AdjAge	0.028	2.35E-02	0.070	7.04E-04	0.010	6.41E-01	-0.001	9.72E-01	-0.010	8.80E-01
AgeAccelPheno	0.123	7.28E-23	0.178	4.45E-18	0.106	1.13E-06	0.078	1.20E-03	0.042	5.22E-01
DNAmTLAdjAge	-0.065	1.91E-07	-0.080	1.17E-04	-0.033	1.36E-01	-0.082	6.05E-04	-0.082	2.11E-01
DNAmAgeSkinBloodClockAdjAge	0.047	1.93E-04	0.102	8.32E-07	0.022	3.17E-01	0.019	4.27E-01	-0.080	2.22E-01

Biweight midcorrelation analyses of EML with chronological age, BMI, AgeAccelHorvath, IEAA, extrinsic epigenetic age acceleration (EEAA) derived from the Hannum clock by up-weighting the contribution of age-related blood cell counts, AgeAccelHannum, AgeAccelGrim, age adjusted DNAm-based surrogate markers of adrenomedullin (DNAmADMAdjAge), beta-2 microglobulin (DNAmB2MAdjAge), cystatin C (DNAmCystatinCAdjAge), growth differentiation factor 15 (DNAmGDF15AdjAge), leptin (DNAmLeptinAdjAge), plasminogen activation inhibitor 1 (DNAmPAI1AdjAge), tissue inhibitor metalloproteinase 1 (DNAmTIMP1AdjAge), smoking pack-years (DNAmPACKYRSAdjAge), AgeAccelPheno, age adjusted DNAm-based surrogate markers of telomere length (DNAmTLAdjAge), and age adjusted SkinBlood clock.

*Meta-analysis using Stouffer's method with weights given by the square root of the number of (non-missing) samples in each data set.

**All analyses except EEAA were adjusted for Age, Sex, Race/ethnicity, Cell types; EEAA was not adjusted for cell types.

Supplementary Table 3. Biweight midcorrelation analysis of EML in FHS, stratified by the direction of acceleration.

Outcome = log(EML) *	Acceleration		Deceleration	
	Bicor r	P_value	Bicor r	P_value
AgeAccelHorvath	0.178	3.81E-09	-0.039	1.71E-01
IEAA	0.150	4.09E-07	-0.064	2.66E-02
EEAA (Unadjusted for cell types)	0.299	4.74E-25	0.089	2.20E-03
AgeAccelHannum	0.239	3.96E-16	0.085	3.12E-03
AgeAccelGrim	0.136	1.64E-05	0.060	2.77E-02
DNAmADMAdjAge	0.149	5.88E-07	0.029	3.14E-01
DNAmB2MAdjAge	0.130	1.90E-05	0.070	1.34E-02
DNAmCystatinCAdjAge	0.135	8.79E-06	-0.026	3.54E-01
DNAmGDF15AdjAge	0.149	1.61E-06	0.078	4.90E-03
DNAmLeptinAdjAge	0.015	6.04E-01	0.068	2.26E-02
DNAmPACKYRSAdjAge	0.077	2.43E-02	0.049	6.15E-02
DNAmPAI1AdjAge	0.075	1.33E-02	-0.035	2.25E-01
DNAmTIMP1AdjAge	0.092	2.17E-03	-0.058	4.30E-02
AgeAccelPheno	0.206	4.49E-12	0.036	2.08E-01
DNAmTLAdjAge	0.064	2.51E-02	-0.127	2.27E-05
DNAmAgeSkinBloodClockAdjAge	0.168	1.40E-08	-0.023	4.17E-01

* Adjusted for Age, Sex, Cell types.

Supplementary Table 4. Stratification analysis: biweight midcorrelation analysis of EML, stratified by sex.

Outcome = log(EML) *	PEG 1				FHS				WHI		JHS			
	Male (n = 126)		Female (n = 111)		Male (n = 1077)		Female (n = 1249)		Femal (n= 2091)		Male (n = 648)		Female (n = 1086)	
	Bicor r	P_value	Bicor r	P_value	Bicor r	P_value	Bicor r	P_value	Bicor r	P_value	Bicor r	P_value	Bicor r	P_value
Age	0.201	2.40E-02	0.200	3.53E-02	0.225	7.53E-14	0.259	1.54E-20	0.104	1.73E-06	0.134	5.98E-04	0.150	7.24E-07
BMI					0.026	3.90E-01	0.025	3.75E-01	-0.009	6.91E-01				
DNAm Age Acceleration														
AgeAccelHorvath	0.105	2.43E-01	0.041	6.71E-01	0.086	4.69E-03	0.126	7.67E-06	0.140	1.34E-10	0.068	8.27E-02	0.082	6.87E-03
IEAA	0.110	2.21E-01	0.033	7.32E-01	0.108	4.02E-04	0.116	4.06E-05	0.144	3.85E-11	0.076	5.19E-02	0.086	4.63E-03
EEAA (Unadjusted for cell types)	0.205	2.13E-02	0.245	9.45E-03	0.305	1.27E-24	0.315	3.35E-30	0.212	1.31E-22	0.180	4.20E-06	0.155	2.90E-07
AgeAccelHannum	0.080	3.73E-01	0.106	2.66E-01	0.215	9.77E-13	0.257	3.02E-20	0.156	6.55E-13	0.158	5.16E-05	0.144	2.05E-06
AgeAccelGrim	0.192	3.13E-02	0.299	1.43E-03	0.218	4.20E-13	0.167	2.71E-09	0.180	9.91E-17	0.082	3.79E-02	0.134	9.43E-06
DNAmADMAdjAge	0.100	2.67E-01	0.223	1.88E-02	0.207	6.85E-12	0.091	1.35E-03	0.070	1.41E-03	-0.024	5.37E-01	0.011	7.06E-01
DNAmB2MAdjAge	0.300	6.40E-04	0.230	1.50E-02	0.238	2.16E-15	0.147	1.94E-07	0.129	3.44E-09	0.063	1.08E-01	0.077	1.13E-02
DNAmCystatinCAdjAge	0.195	2.86E-02	0.187	4.89E-02	0.181	2.42E-09	0.075	7.81E-03	0.241	6.48E-29	0.039	3.25E-01	0.105	5.43E-04
DNAmGDF15AdjAge	0.129	1.49E-01	0.172	7.03E-02	0.211	2.85E-12	0.134	2.06E-06	0.127	5.80E-09	0.097	1.37E-02	0.167	2.91E-08
DNAmLeptinAdjAge	-0.073	4.19E-01	0.138	1.49E-01	0.071	1.94E-02	-0.037	1.89E-01	-0.035	1.11E-01	0.043	2.75E-01	0.046	1.27E-01
DNAmPACKYRSAdjAge	0.184	3.89E-02	0.120	2.10E-01	0.161	1.06E-07	0.179	2.09E-10	0.172	2.23E-15	0.113	4.05E-03	0.147	1.19E-06
DNAmPAI1AdjAge	0.026	7.70E-01	0.362	9.25E-05	0.028	3.66E-01	0.039	1.65E-01	-0.004	8.59E-01	0.053	1.76E-01	0.065	3.21E-02
DNAmTIMP1AdjAge	-0.038	6.72E-01	0.031	7.50E-01	0.100	1.07E-03	0.060	3.41E-02	0.010	6.41E-01	-0.031	4.27E-01	0.016	5.97E-01
AgeAccelPheno	0.123	1.68E-01	-0.080	4.02E-01	0.211	2.66E-12	0.152	6.90E-08	0.106	1.13E-06	0.079	4.47E-02	0.080	8.40E-03
DNAmTLAdjAge	-0.052	5.64E-01	-0.166	8.26E-02	-0.061	4.61E-02	-0.101	3.63E-04	-0.033	1.36E-01	-0.088	2.56E-02	-0.069	2.21E-02
DNAmAgeSkinBloodClockAdjAge	-0.029	7.45E-01	-0.141	1.40E-01	0.105	5.37E-04	0.102	2.90E-04	0.022	3.17E-01	-0.010	7.99E-01	0.032	2.88E-01
Cell types														

CD8.naive	0.043	6.32E-01	0.044	6.47E-01	-0.076	1.27E-02	-0.068	1.62E-02	0.020	3.66E-01	-0.008	8.30E-01	-0.010	7.32E-01
CD8pCD28nCD45RAn	0.018	8.44E-01	0.179	6.00E-02	0.076	1.26E-02	0.096	7.00E-04	0.086	8.16E-05	0.068	8.43E-02	0.030	3.28E-01
PlasmaBlast	-0.089	3.19E-01	-0.125	1.92E-01	-0.012	6.90E-01	-0.088	1.81E-03	-0.070	1.39E-03	-0.148	1.60E-04	-0.139	4.57E-06
CD4T	-0.134	1.35E-01	-0.133	1.65E-01	-0.150	7.97E-07	-0.143	4.03E-07	-0.113	2.17E-07	-0.049	2.17E-01	-0.120	7.55E-05
Gran	-0.182	4.18E-02	-0.128	1.80E-01	-0.065	3.37E-02	-0.084	2.81E-03	-0.016	4.74E-01	-0.065	9.80E-02	-0.111	2.57E-04

* All analyses except EEAA were adjusted for Age, Sex, Race/ethnicity, Cell types; EEAA was not adjusted for cell types.

Supplementary Table 5. Sensitivity analysis: biweight midcorrelation analysis of EML, additionally adjusted for age^2.

Outcome = log(EML)	PEG 1 (n = 237)				FHS (n = 2326)				WHI (n= 2091)				JHS (n= 1734)			
	Model 1 *		Model 2 **		Model 1 *		Model 2 **		Model 1 *		Model 2 **		Model 1 *		Model 2 **	
	Bicor r	P_value	Bicor r	P_value	Bicor r	P_value	Bicor r	P_value	Bicor r	P_value	Bicor r	P_value	Bicor r	P_value	Bicor r	P_value
AgeAccelHorvath	0.071	2.75E-01	0.089	1.72E-01	0.106	3.11E-07	0.106	3.08E-07	0.140	1.34E-10	0.141	9.63E-11	0.079	9.68E-04	0.100	2.84E-05
IEAA	0.073	2.63E-01	0.091	1.61E-01	0.109	1.26E-07	0.109	1.23E-07	0.144	3.85E-11	0.144	3.19E-11	0.080	8.50E-04	0.102	2.18E-05
EEAA (Unadjusted for cell types)	0.211	1.05E-03	0.225	4.80E-04	0.297	1.12E-48	0.298	7.40E-49	0.212	1.31E-22	0.210	2.35E-22	0.166	3.09E-12	0.168	2.13E-12
AgeAccelHannum	0.095	1.46E-01	0.111	8.81E-02	0.225	4.12E-28	0.225	3.79E-28	0.156	6.55E-13	0.156	8.30E-13	0.148	6.33E-10	0.158	3.69E-11
AgeAccelGrim	0.224	5.23E-04	0.219	7.01E-04	0.173	3.74E-17	0.173	3.87E-17	0.180	9.91E-17	0.180	1.09E-16	0.111	3.46E-06	0.118	8.87E-07
DNAmADMAdjAge	0.157	1.58E-02	0.156	1.63E-02	0.121	4.58E-09	0.121	4.65E-09	0.070	1.41E-03	0.069	1.56E-03	-0.017	4.71E-01	-0.019	4.34E-01
DNAmB2MAdjAge	0.287	7.20E-06	0.288	6.61E-06	0.189	4.69E-20	0.188	4.84E-20	0.129	3.44E-09	0.128	4.33E-09	0.074	2.05E-03	0.072	2.75E-03
DNAmCystatinCAdjAge	0.183	4.64E-03	0.182	4.96E-03	0.119	8.71E-09	0.119	8.84E-09	0.241	6.48E-29	0.240	8.64E-29	0.081	7.30E-04	0.075	1.69E-03
DNAmGDF15AdjAge	0.162	1.27E-02	0.162	1.26E-02	0.167	6.09E-16	0.167	6.12E-16	0.127	5.80E-09	0.126	7.40E-09	0.142	3.01E-09	0.143	2.11E-09
DNAmLeptinAdjAge	0.063	3.34E-01	0.061	3.46E-01	-0.021	3.19E-01	-0.021	3.23E-01	-0.035	1.11E-01	-0.034	1.20E-01	-0.031	2.03E-01	-0.031	2.02E-01
DNAmPACKYRSAdjAge	0.150	2.13E-02	0.144	2.64E-02	0.166	7.99E-16	0.166	8.60E-16	0.172	2.23E-15	0.173	1.67E-15	0.137	1.10E-08	0.148	6.41E-10
DNAmPAI1AdjAge	0.173	7.56E-03	0.170	8.84E-03	0.024	2.49E-01	0.024	2.48E-01	-0.004	8.59E-01	-0.001	9.57E-01	0.061	1.07E-02	0.067	5.03E-03
DNAmTIMP1AdjAge	-0.010	8.80E-01	-0.008	9.08E-01	0.070	7.04E-04	0.070	7.11E-04	0.010	6.41E-01	0.008	7.01E-01	-0.001	9.72E-01	-0.002	9.42E-01
AgeAccelPheno	0.042	5.22E-01	0.051	4.36E-01	0.178	4.45E-18	0.178	4.22E-18	0.106	1.13E-06	0.107	9.47E-07	0.078	1.20E-03	0.083	5.33E-04
DNAmTLAdjAge	-0.082	2.11E-01	-0.087	1.84E-01	-0.080	1.17E-04	-0.080	1.16E-04	-0.033	1.36E-01	-0.033	1.30E-01	-0.082	6.05E-04	-0.093	1.08E-04
DNAmAgeSkinBloodClockAdjAge	-0.080	2.22E-01	-0.045	4.91E-01	0.102	8.32E-07	0.102	7.97E-07	0.022	3.17E-01	0.024	2.79E-01	0.019	4.27E-01	0.047	5.25E-02

* Adjusted (Age, Sex, Race/ethnicity, Cell types)

** Adjusted (Age, Age2, Sex, Race/ethnicity, Cell types)

Supplementary Table 6. Sensitivity analysis: biweight midcorrelation analysis of EML, additionally adjusted for BMI.

Outcome = log(EML)	FHS (n = 2326)				WHI (n= 2091)			
	Model 1 *		Model 2 **		Model 1 *		Model 2 **	
	Bicor r	P_value	Bicor r	P_value	Bicor r	P_value	Bicor r	P_value
AgeAccelHorvath	0.106	3.11E-07	0.110	1.16E-07	0.140	1.34E-10	0.144	4.13E-11
IEAA	0.109	1.26E-07	0.113	4.37E-08	0.144	3.85E-11	0.148	1.36E-11
EEAA (Unadjusted for cell types)	0.297	1.12E-48	0.298	1.29E-48	0.212	1.31E-22	0.216	2.92E-23
AgeAccelHannum	0.225	4.12E-28	0.226	3.19E-28	0.156	6.55E-13	0.157	5.85E-13
AgeAccelGrim	0.173	3.74E-17	0.172	8.56E-17	0.180	9.91E-17	0.181	1.02E-16
DNAmADMAdjAge	0.121	4.58E-09	0.123	2.93E-09	0.070	1.41E-03	0.074	7.79E-04
DNAmB2MAdjAge	0.189	4.69E-20	0.190	2.84E-20	0.129	3.44E-09	0.130	2.44E-09
DNAmCystatinCAdjAge	0.119	8.71E-09	0.117	1.51E-08	0.241	6.48E-29	0.244	1.31E-29
DNAmGDF15AdjAge	0.167	6.09E-16	0.165	1.37E-15	0.127	5.80E-09	0.127	7.21E-09
DNAmLeptinAdjAge	-0.021	3.19E-01	-0.021	3.21E-01	-0.035	1.11E-01	-0.029	1.93E-01
DNAmPACKYRSAdjAge	0.166	7.99E-16	0.164	1.88E-15	0.172	2.23E-15	0.169	1.09E-14
DNAmPAI1AdjAge	0.024	2.49E-01	0.021	3.16E-01	-0.004	8.59E-01	0.007	7.34E-01
DNAmTIMP1AdjAge	0.070	7.04E-04	0.068	1.03E-03	0.010	6.41E-01	0.016	4.71E-01

AgeAccelPheno	0.178	4.45E-18	0.180	2.10E-18	0.106	1.13E-06	0.111	4.26E-07
DNAmTLAdjAge	-0.080	1.17E-04	-0.079	1.47E-04	-0.033	1.36E-01	-0.033	1.35E-01
DNAmAgeSkinBloodClockAdjAge	0.102	8.32E-07	0.104	5.72E-07	0.022	3.17E-01	0.024	2.71E-01

* Adjusted (Age, Sex, Race/ethnicity, Cell types)
** Adjusted (Age, Sex, Race/ethnicity, Cell types, BMI)

Supplementary Table 7. Sensitivity analysis: Biweight midcorrelation analysis of EML in FHS using different SEM cutoffs in FHS.

Outcome = log(EML) *	EML (3IQR)		EML (2IQR) **		EML (4IQR) ***	
	Bicor r	P_value	Bicor r	P_value	Bicor r	P_value
Age	0.244	7.15E-33	0.223	1.02E-27	0.247	1.13E-33
BMI	0.017	4.23E-01	0.006	7.60E-01	0.019	3.49E-01
DNAm Age Acceleration						
AgeAccelHorvath	0.106	3.11E-07	0.073	4.38E-04	0.112	5.44E-08
IEAA	0.109	1.26E-07	0.077	2.00E-04	0.135	5.28E-11
EEAA (Unadjusted for cell types)	0.297	1.12E-48	0.281	1.89E-43	0.293	2.46E-47
AgeAccelHannum	0.225	4.12E-28	0.224	5.97E-28	0.204	1.98E-23
AgeAccelGrim	0.173	3.74E-17	0.147	1.03E-12	0.180	1.75E-18
DNAmADMAdjAge	0.121	4.58E-09	0.084	4.41E-05	0.133	1.31E-10
DNAmB2MAdjAge	0.189	4.69E-20	0.178	3.79E-18	0.180	2.28E-18
DNAmCystatinCAdjAge	0.119	8.71E-09	0.116	2.03E-08	0.111	6.85E-08
DNAmGDF15AdjAge	0.167	6.09E-16	0.157	2.52E-14	0.161	4.38E-15
DNAmLeptinAdjAge	-0.021	3.19E-01	-0.019	3.62E-01	-0.027	1.97E-01
DNAmPACKYRSAdjAge	0.166	7.99E-16	0.142	5.64E-12	0.176	1.05E-17
DNAmPAI1AdjAge	0.024	2.49E-01	0.005	8.17E-01	0.037	7.75E-02
DNAmTIMP1AdjAge	0.070	7.04E-04	0.068	9.46E-04	0.071	6.02E-04
AgeAccelPheno	0.178	4.45E-18	0.173	3.96E-17	0.164	1.37E-15
DNAmTLAdjAge	-0.080	1.17E-04	-0.068	1.04E-03	-0.073	4.37E-04
DNAmAgeSkinBloodClockAdjAge	0.102	8.32E-07	0.072	5.20E-04	0.116	1.77E-08
Cell types						
CD8.naive	-0.072	5.20E-04	-0.038	6.97E-02	-0.084	4.77E-05
CD8pCD28nCD45RAn	0.085	3.90E-05	0.112	6.64E-08	0.038	6.37E-02
PlasmaBlast	-0.054	8.94E-03	-0.096	3.13E-06	-0.021	3.10E-01
CD4T	-0.146	1.68E-12	-0.176	9.14E-18	-0.149	5.34E-13
Gran	-0.075	2.72E-04	-0.074	3.37E-04	-0.066	1.52E-03

* Adjusted for age, sex, cell types.
** SEMs (2IQR) were defined as DNA methylation mutations that were greater than 2 times the IQR above the upper quartile of a given CpG or less than 2 times the IQR below the lower quartile of a given CpG.
*** SEMs (4IQR) were defined as DNA methylation mutations that were greater than 4 times the IQR above the upper quartile of a given CpG or less than 4 times the IQR below the lower quartile of a given CpG.

Supplementary Table 8. Sensitivity analysis: biweight midcorrelation analysis of EML derived from methylation data with various normalization methods (Illumina, Noob and Quantile normalization).

	PEG 1 (n = 237)							
Outcome = log(EML)	Illumina		Functional		Noob		Quantile	
	Bicor r	P_value	Bicor r	P_value	Bicor r	P_value	Bicor r	P_value
**Adjusted (Age, Sex, Race/ethnicity, Cell types)**								
AgeAccelHorvath	0.075	2.49E-01	0.070	2.82E-01	0.077	2.40E-01	0.021	7.50E-01
IEAA	0.080	2.17E-01	0.067	3.07E-01	0.074	2.59E-01	0.029	6.55E-01
AgeAccelHannum	0.109	9.29E-02	0.093	1.52E-01	0.104	1.10E-01	0.036	5.86E-01
AgeAccelGrim	0.203	1.71E-03	0.213	9.69E-04	0.241	1.82E-04	0.134	3.93E-02
Cell types								
CD8.naive	0.070	2.84E-01	0.042	5.23E-01	0.029	6.52E-01	0.066	3.10E-01
CD8pCD28nCD45RAn	0.063	3.34E-01	0.082	2.07E-01	0.097	1.36E-01	0.074	2.58E-01
PlasmaBlast	-0.104	1.11E-01	-0.110	9.23E-02	-0.111	8.91E-02	-0.068	2.98E-01
CD4T	-0.080	2.20E-01	-0.118	6.95E-02	-0.115	7.66E-02	0.018	7.85E-01
Gran	-0.152	1.90E-02	-0.170	8.67E-03	-0.163	1.21E-02	-0.013	8.48E-01

Supplementary Table 9. SEMs enriched within clock CpGs.

	FHS (n = 2326)	WHI (n= 2091)	JHS (n= 1734)	PEG 1 (n = 237)
**Clock**	**N**	**N**	**N**	**N**
**HorvathClock (353 CpGs)**				
Significant	46	34	22	4
Non-Significant	2280	2057	1712	233
**PhenoClock (513 CpGs)**				
Significant	151	97	121	19
Non-Significant	2175	1994	1613	218
**HannumClock (71 CpGs)**				
Significant	26	10	19	5
Non-Significant	2300	2081	1715	232

Supplementary Table 10. SEMs enriched within gene regions

	FHS (n = 2326)	WHI (n= 2091)	JHS (n= 1734)	PEG 1 (n = 237)
**Genomic region**	**N**	**N**	**N**	**N**
**TSS1500**				
Significant	545	110	229	23
Non-Significant	1781	1981	1505	214
**TSS200**				
Significant	908	385	372	142
Non-Significant	1418	1706	1362	95
**5'UTR**				
Significant	213	71	64	8
Non-Significant	2113	2020	1670	229
**1stExon**				
Significant	595	346	322	84
Non-Significant	1731	1745	1412	153
**Gene Body**				
Significant	296	693	357	24
Non-Significant	2030	1398	1377	213
**3'UTR**				
Significant	110	276	179	6
Non-Significant	2216	1815	1555	231

Supplementary Table 11. SEMs enriched within regulatory regions.

	JHS (n= 1734)
**Genomic region**	**N**
**Enhancer from FANTOM5**	
Significant	54
Non-Significant	1680
**DNase hypersensitive from ENCODE**	
Significant	284
Non-Significant	1450
**Open chromatin from ENCODE**	
Significant	436
Non-Significant	1298
**Transcription factor binding site from ENCODE**	
Significant	383
Non-Significant	1351
**Promoter from Methylation Consortium**	
Significant	165
Non-Significant	1578

Supplementary Table 12. Top SEMs enriched KEGG pathways significantly associated with faster AgeAccelHorvath.

	KEGG Pathway	Description	Number of people with SEMs enriched	AgeAccel_Coef *	AgeAccel_Pvalue
**FHS (n = 2326)**	hsa05165	Human papillomavirus infection	355	1.664	7.62E-07
	hsa04360	Axon guidance	347	1.726	8.53E-07
	hsa05032	Morphine addiction	346	2.138	1.13E-11
	hsa04713	Circadian entrainment	341	1.796	4.97E-08
	hsa05033	Nicotine addiction	339	2.239	4.33E-13
	hsa04510	Focal adhesion	332	1.684	1.25E-06
	hsa04080	Neuroactive ligand-receptor interaction	326	2.487	2.84E-15
	hsa04724	Glutamatergic synapse	300	2.412	7.38E-13
	hsa04934	Cushing syndrome	297	2.041	1.15E-08
	hsa04727	GABAergic synapse	294	2.419	7.00E-13
**WHI (n= 2091)**	hsa00053	Ascorbate and aldarate metabolism	312	0.800	1.88E-02
	hsa04514	Cell adhesion molecules (CAMs)	237	1.255	8.19E-04
	hsa04724	Glutamatergic synapse	231	1.220	5.95E-03
	hsa04360	Axon guidance	229	2.071	8.38E-06
	hsa04713	Circadian entrainment	208	2.083	4.77E-06
	hsa05166	Human T-cell leukemia virus 1 infection	193	-1.660	5.10E-05
	hsa05032	Morphine addiction	189	2.541	1.45E-08
	hsa04934	Cushing syndrome	171	1.504	3.23E-03
	hsa04921	Oxytocin signaling pathway	167	1.445	6.29E-03
	hsa04020	Calcium signaling pathway	165	2.235	8.79E-06
**JHS (n= 1734)**	hsa05032	Morphine addiction	402	1.167	9.84E-04
	hsa04713	Circadian entrainment	395	0.830	2.73E-02
	hsa04921	Oxytocin signaling pathway	372	0.798	3.33E-02
	hsa04020	Calcium signaling pathway	327	1.258	8.01E-04
	hsa04512	ECM-receptor interaction	317	1.032	1.44E-02
	hsa04727	GABAergic synapse	282	1.093	9.28E-03
	hsa04934	Cushing syndrome	249	1.370	1.00E-03
	hsa04014	Ras signaling pathway	245	0.957	3.90E-02
	hsa05412	Arrhythmogenic right ventricular cardiomyopathy (ARVC)	227	1.690	1.44E-04
	hsa04261	Adrenergic signaling in cardiomyocytes	226	1.006	2.95E-02

	hsa04360	Axon guidance	59	0.319	9.06E-01
	hsa04713	Circadian entrainment	52	0.226	9.06E-01
	hsa05224	Breast cancer	46	0.586	8.45E-01
	hsa05165	Human papillomavirus infection	45	2.202	3.04E-01
**PEG 1 (n = 237)**	hsa05032	Morphine addiction	44	0.182	9.21E-01
	hsa05226	Gastric cancer	44	0.868	7.78E-01
	hsa04934	Cushing syndrome	43	0.907	7.78E-01
	hsa04390	Hippo signaling pathway	39	0.916	7.78E-01
	hsa04724	Glutamatergic synapse	39	1.630	5.97E-01
	hsa04015	Rap1 signaling pathway	37	-0.390	8.94E-01

* Age acceleration residual as dependent variable, significant enrichment as independent variable, adjusted for nlog(EML)

Supplementary Table 13. Top SEMs enriched KEGG pathways significantly associated with faster AgeAccelHannum

	KEGG Pathway	Description	Number of people with SEMs enriched	AgeAccel_Coef *	AgeAccel_Pvalue
	hsa05165	Human papillomavirus infection	355	1.143	1.14E-03
	hsa04360	Axon guidance	347	0.792	3.98E-02
	hsa05032	Morphine addiction	346	2.222	1.67E-12
	hsa04713	Circadian entrainment	341	1.959	2.24E-09
**FHS (n = 2326)**	hsa05033	Nicotine addiction	339	2.083	1.69E-11
	hsa04510	Focal adhesion	332	1.074	3.35E-03
	hsa04080	Neuroactive ligand-receptor interaction	326	2.262	1.13E-12
	hsa04724	Glutamatergic synapse	300	2.029	1.84E-09
	hsa04934	Cushing syndrome	297	2.283	1.57E-10
	hsa00053	Ascorbate and aldarate metabolism	296	0.917	5.61E-03
	hsa04724	Glutamatergic synapse	231	1.461	5.59E-04
	hsa04360	Axon guidance	229	1.477	1.19E-03
	hsa04713	Circadian entrainment	208	1.987	7.57E-06
	hsa05166	Human T-cell leukemia virus 1 infection	193	-1.133	5.13E-03
**WHI (n= 2091)**	hsa05032	Morphine addiction	189	2.373	5.72E-08
	hsa04934	Cushing syndrome	171	2.160	9.38E-06
	hsa04921	Oxytocin signaling pathway	167	1.330	9.42E-03
	hsa04020	Calcium signaling pathway	165	1.844	1.77E-04
	hsa04510	Focal adhesion	145	-1.175	4.02E-02
	hsa04024	cAMP signaling pathway	137	2.147	3.52E-05
	hsa04360	Axon guidance	451	0.630	3.43E-02
	hsa05032	Morphine addiction	402	0.927	5.24E-04
	hsa04713	Circadian entrainment	395	0.582	3.85E-02
	hsa04020	Calcium signaling pathway	327	0.898	1.62E-03
**JHS (n= 1734)**	hsa04512	ECM-receptor interaction	317	0.841	6.33E-03
	hsa04727	GABAergic synapse	282	1.503	4.07E-08
	hsa05165	Human papillomavirus infection	277	0.685	3.43E-02
	hsa04934	Cushing syndrome	249	1.445	9.84E-07
	hsa04024	cAMP signaling pathway	239	0.906	7.88E-03
	hsa05412	Arrhythmogenic right ventricular cardiomyopathy (ARVC)	227	0.865	1.91E-02
	hsa04360	Axon guidance	59	0.418	8.86E-01
	hsa04713	Circadian entrainment	52	1.182	7.74E-01
	hsa05224	Breast cancer	46	-0.554	8.86E-01
**PEG 1 (n = 237)**	hsa05165	Human papillomavirus infection	45	0.654	8.68E-01
	hsa05032	Morphine addiction	44	0.060	9.81E-01
	hsa05226	Gastric cancer	44	0.345	8.86E-01
	hsa04934	Cushing syndrome	43	0.820	8.66E-01

hsa04390	Hippo signaling pathway	39	0.524	8.86E-01
hsa04724	Glutamatergic synapse	39	1.877	6.22E-01
hsa04015	Rap1 signaling pathway	37	-0.117	9.61E-01

[*] Age acceleration residual as dependent variable, significant enrichment as independent variable, adjusted for nlog(EML)

Supplementary Table 14. Top SEMs enriched KEGG pathways significantly associated with faster IEAA.

	KEGG Pathway	Description	Number of people with SEMs enriched	AgeAccel_Coef [*]	AgeAccel_Pvalue
**FHS (n = 2326)**	hsa05165	Human papillomavirus infection	355	0.887	2.56E-02
	hsa05032	Morphine addiction	346	1.055	3.76E-03
	hsa04713	Circadian entrainment	341	0.939	1.50E-02
	hsa05033	Nicotine addiction	339	1.560	3.24E-06
	hsa04080	Neuroactive ligand-receptor interaction	326	1.678	1.16E-06
	hsa04724	Glutamatergic synapse	300	1.392	2.08E-04
	hsa04934	Cushing syndrome	297	1.055	1.22E-02
	hsa04727	GABAergic synapse	294	1.400	2.06E-04
	hsa04020	Calcium signaling pathway	292	1.850	7.65E-07
	hsa05224	Breast cancer	291	1.021	1.53E-02
**WHI (n= 2091)**	hsa04724	Glutamatergic synapse	231	1.048	1.74E-02
	hsa04360	Axon guidance	229	1.439	2.09E-03
	hsa04713	Circadian entrainment	208	1.645	2.88E-04
	hsa05166	Human T-cell leukemia virus 1 infection	193	-1.187	4.08E-03
	hsa05032	Morphine addiction	189	2.108	3.21E-06
	hsa04921	Oxytocin signaling pathway	167	1.072	4.54E-02
	hsa04020	Calcium signaling pathway	165	1.789	3.44E-04
	hsa04015	Rap1 signaling pathway	163	1.306	1.63E-02
	hsa04024	cAMP signaling pathway	137	2.296	1.60E-05
	hsa05033	Nicotine addiction	135	2.201	1.11E-05
**JHS (n= 1734)**	hsa05032	Morphine addiction	402	0.884	3.66E-02
	hsa04934	Cushing syndrome	249	1.005	4.40E-02
	hsa05412	Arrhythmogenic right ventricular cardiomyopathy (ARVC)	227	1.322	8.85E-03
	hsa05033	Nicotine addiction	222	1.367	3.57E-03
	hsa05226	Gastric cancer	166	1.293	2.91E-02
	hsa04080	Neuroactive ligand-receptor interaction	164	2.127	2.41E-05
	hsa05414	Dilated cardiomyopathy (DCM)	155	1.893	2.23E-04
	hsa04721	Synaptic vesicle cycle	115	2.110	2.54E-04
	hsa05410	Hypertrophic cardiomyopathy (HCM)	108	2.627	1.26E-05
	hsa04950	Maturity onset diabetes of the young	81	1.958	8.00E-03
**PEG 1 (n = 237)**	hsa04360	Axon guidance	59	-0.324	9.32E-01
	hsa04713	Circadian entrainment	52	-0.461	9.10E-01
	hsa05224	Breast cancer	46	-0.076	9.81E-01
	hsa05165	Human papillomavirus infection	45	2.479	1.66E-01
	hsa05032	Morphine addiction	44	-0.302	9.32E-01
	hsa05226	Gastric cancer	44	0.063	9.81E-01
	hsa04934	Cushing syndrome	43	0.413	9.32E-01
	hsa04390	Hippo signaling pathway	39	0.472	9.10E-01
	hsa04724	Glutamatergic synapse	39	1.412	7.33E-01
	hsa04015	Rap1 signaling pathway	37	-1.111	8.06E-01

[*] Age acceleration residual as dependent variable, significant enrichment as independent variable, adjusted for nlog(EML)

Supplementary Table 15. Top SEMs enriched KEGG pathways significantly associated with faster AgeAccelGrim.

	KEGG Pathway	Description	Number of people with SEMs enriched	AgeAccel_Coef *	AgeAccel_Pvalue
**FHS (n = 2326)**	hsa04360	Axon guidance	347	-0.798	4.97E-02
	hsa04510	Focal adhesion	332	-0.855	3.24E-02
	hsa04080	Neuroactive ligand-receptor interaction	326	-0.742	4.31E-02
	hsa04390	Hippo signaling pathway	284	-1.431	5.72E-04
	hsa04151	PI3K-Akt signaling pathway	265	-0.956	2.56E-02
	hsa05205	Proteoglycans in cancer	263	-1.454	8.02E-04
	hsa05226	Gastric cancer	256	-1.399	1.04E-03
	hsa04512	ECM-receptor interaction	252	-0.865	3.81E-02
	hsa04550	Signaling pathways regulating pluripotency of stem cells	249	-1.598	1.57E-04
	hsa04010	MAPK signaling pathway	240	-1.168	9.80E-03
**WHI (n= 2091)**	hsa04510	Focal adhesion	145	-1.069	2.69E-02
	hsa04010	MAPK signaling pathway	135	-1.251	1.37E-02
	hsa05033	Nicotine addiction	135	0.969	2.45E-02
	hsa04080	Neuroactive ligand-receptor interaction	130	1.157	8.32E-03
	hsa04072	Phospholipase D signaling pathway	129	-1.556	1.20E-03
	hsa04310	Wnt signaling pathway	117	-1.251	1.72E-02
	hsa04152	AMPK signaling pathway	111	-1.792	4.40E-04
	hsa04911	Insulin secretion	102	1.178	1.86E-02
	hsa04014	Ras signaling pathway	95	-1.463	1.03E-02
	hsa04144	Endocytosis	91	-1.763	1.12E-03
**JHS (n= 1734)**	hsa04724	Glutamatergic synapse	573	-0.148	9.05E-01
	hsa04360	Axon guidance	451	0.254	8.92E-01
	hsa04725	Cholinergic synapse	437	0.164	9.05E-01
	hsa04510	Focal adhesion	413	-0.163	9.07E-01
	hsa05032	Morphine addiction	402	0.352	7.97E-01
	hsa00053	Ascorbate and aldarate metabolism	400	0.093	9.38E-01
	hsa04713	Circadian entrainment	395	0.240	8.92E-01
	hsa04015	Rap1 signaling pathway	391	0.228	8.95E-01
	hsa00040	Pentose and glucuronate interconversions	374	-0.038	9.62E-01
	hsa04080	Neuroactive ligand-receptor interaction	164	1.793	9.56E-03
**PEG 1 (n = 237)**	hsa04360	Axon guidance	59	-0.468	9.20E-01
	hsa04713	Circadian entrainment	52	-0.521	9.20E-01
	hsa05224	Breast cancer	46	-2.263	4.84E-01
	hsa05165	Human papillomavirus infection	45	-1.114	8.36E-01
	hsa05032	Morphine addiction	44	-0.895	9.20E-01
	hsa05226	Gastric cancer	44	-1.497	6.67E-01
	hsa04934	Cushing syndrome	43	0.378	9.20E-01
	hsa04390	Hippo signaling pathway	39	-0.786	9.20E-01
	hsa04724	Glutamatergic synapse	39	0.526	9.20E-01
	hsa04015	Rap1 signaling pathway	37	-1.882	6.67E-01

* Age acceleration residual as dependent variable, significant enrichment as independent variable, adjusted for nlog(EML)

Supplementary Table 16. Top SEMs enriched KEGG pathways significantly associated with faster AgeAccelPheno.

	KEGG Pathway	Description	Number of people with SEMs enriched	AgeAccel_Coef *	AgeAccel_Pvalue
**FHS (n = 2326)**	hsa05165	Human papillomavirus infection	355	0.003	1.00E+00
	hsa04360	Axon guidance	347	-1.074	2.14E-01
	hsa05032	Morphine addiction	346	0.409	8.14E-01
	hsa04713	Circadian entrainment	341	0.078	9.62E-01
	hsa05033	Nicotine addiction	339	0.300	8.43E-01
	hsa04510	Focal adhesion	332	0.049	9.74E-01
	hsa04080	Neuroactive ligand-receptor interaction	326	0.120	9.13E-01
	hsa04724	Glutamatergic synapse	300	-0.022	9.84E-01
	hsa04934	Cushing syndrome	297	-0.255	8.99E-01
	hsa00053	Ascorbate and aldarate metabolism	296	0.168	9.02E-01
**WHI (n= 2091)**	hsa04940	Type I diabetes mellitus	493	-0.525	4.15E-01
	hsa05330	Allograft rejection	337	-0.167	8.74E-01
	hsa05332	Graft-versus-host disease	313	-0.344	6.60E-01
	hsa00053	Ascorbate and aldarate metabolism	312	0.285	7.38E-01
	hsa00040	Pentose and glucuronate interconversions	305	0.127	9.19E-01
	hsa05416	Viral myocarditis	299	-0.086	9.67E-01
	hsa00860	Porphyrin and chlorophyll metabolism	272	0.048	9.81E-01
	hsa05320	Autoimmune thyroid disease	272	-0.228	8.32E-01
	hsa04612	Antigen processing and presentation	257	-0.228	8.32E-01
	hsa00980	Metabolism of xenobiotics by cytochrome P450	253	-0.017	9.88E-01
**JHS (n= 1734)**	hsa04724	Glutamatergic synapse	573	0.029	9.58E-01
	hsa04360	Axon guidance	451	0.290	8.08E-01
	hsa04725	Cholinergic synapse	437	0.150	8.98E-01
	hsa04510	Focal adhesion	413	-0.604	5.25E-01
	hsa05032	Morphine addiction	402	0.785	3.30E-01
	hsa00053	Ascorbate and aldarate metabolism	400	0.464	5.82E-01
	hsa04713	Circadian entrainment	395	1.052	1.57E-01
	hsa04080	Neuroactive ligand-receptor interaction	164	2.014	3.77E-02
	hsa05414	Dilated cardiomyopathy (DCM)	155	2.054	3.77E-02
	hsa05410	Hypertrophic cardiomyopathy (HCM)	108	2.306	4.22E-02
**PEG 1 (n = 237)**	hsa04360	Axon guidance	59	-0.606	9.29E-01
	hsa04713	Circadian entrainment	52	0.351	9.53E-01
	hsa05224	Breast cancer	46	-1.177	9.29E-01
	hsa05165	Human papillomavirus infection	45	0.292	9.62E-01
	hsa05032	Morphine addiction	44	-0.684	9.29E-01
	hsa05226	Gastric cancer	44	0.589	9.29E-01
	hsa04934	Cushing syndrome	43	0.075	9.86E-01
	hsa04390	Hippo signaling pathway	39	-2.425	7.03E-01
	hsa04724	Glutamatergic synapse	39	1.064	9.29E-01
	hsa04015	Rap1 signaling pathway	37	-0.464	9.29E-01

* Age acceleration residual as dependent variable, significant enrichment as independent variable, adjusted for nlog(SEM)

Supplementary Table 17. Association between Clock region-specific EML and corresponding DNAm AgeAccel.

Outcome = log(clock EML)	PEG 1 (n = 237)		FHS (n = 2326)		WHI (n= 2091)		JHS (n= 1734)	
Model *	Bicor r	P_value	Bicor r	P_value	Bicor r	P_value	Bicor r	P_value
HorvathClock (353 CpGs) v. AgeAccel	0.008	9.07E-01	0.020	8.35E-01	0.034	1.23E-01	0.180	4.16E-14
PhenoClock (513 CpGs) v. AgeAccelPheno	0.008	9.03E-01	0.013	5.29E-01	0.058	8.26E-03	0.064	7.57E-03
HannumClock (71CpGs) v. AgeAccelHannum	0.071	2.77E-01	-0.030	1.51E-01	0.017	4.28E-01	0.044	6.84E-02

* Adjusted for Age, Sex, Cell types, Race/ethnicity, Log(total EML)

Supplementary Table 18. Biweight midcorrelation analysis of regulatory region-specific EML in JHS.

Outcome = log(Region EML) *	Enhancer (CpGs = 26395)		DNAse (CpGs = 466862)		Open Chromatin (CpGs = 108758)		TFBS (CpGs = 122647)		Promoter (CpGs = 110008)	
	Bicor r	P_value	Bicor r	P_value	Bicor r	P_value	Bicor r	P_value	Bicor r	P_value
Age	-0.045	6.01E-02	0.169	1.24E-12	0.019	4.34E-01	-0.065	7.11E-03	-0.027	2.58E-01
DNAm Age Acceleration										
AgeAccelHorvath	-0.027	2.66E-01	0.129	6.75E-08	0.026	2.73E-01	-0.012	6.14E-01	-0.003	8.94E-01
IEAA	-0.027	2.64E-01	0.132	3.26E-08	0.025	3.04E-01	-0.023	3.34E-01	-0.002	9.32E-01
AgeAccelHannum	-0.059	1.36E-02	0.236	2.08E-23	0.002	9.26E-01	-0.089	1.94E-04	-0.029	2.35E-01
AgeAccelGrim	0.022	3.66E-01	0.098	4.01E-05	0.018	4.56E-01	-0.038	1.17E-01	-0.013	5.99E-01
Cell types										
CD8.naive	-0.004	8.69E-01	-0.101	2.40E-05	0.029	2.20E-01	0.027	2.67E-01	-0.029	2.23E-01
CD8pCD28nCD45RAn	0.016	5.10E-01	0.073	2.33E-03	-0.004	8.57E-01	-0.024	3.21E-01	0.038	1.12E-01
PlasmaBlast	-0.020	3.98E-01	-0.011	6.41E-01	0.038	1.11E-01	0.016	4.99E-01	-0.040	9.44E-02
CD4T	-0.012	6.05E-01	-0.090	1.79E-04	0.086	3.33E-04	0.109	5.20E-06	0.016	5.03E-01
Gran	-0.067	5.15E-03	-0.181	3.39E-14	0.106	8.95E-06	0.151	2.47E-10	-0.049	4.21E-02

* Adjusted for Age, Sex, Cell types, Log(total EML).

Supplementary Table 19. Biweight midcorrelation analysis of EML in FHS, stratified by the direction of SEM.

Outcome = log(EML) *	All EML		Hypermethylated EML		Hypomethylated EML	
	Bicor r	P_value	Bicor r	P_value	Bicor r	P_value
Age	0.244	7.15E-33	0.271	1.81E-40	0.139	1.53E-11
BMI	0.017	4.23E-01	0.016	4.49E-01	0.012	5.53E-01
DNAm Age Acceleration						
AgeAccelHorvath	0.106	3.11E-07	0.101	1.19E-06	0.092	9.46E-06
IEAA	0.109	1.26E-07	0.100	1.34E-06	0.107	2.22E-07
EEAA (Unadjusted for cell types)	0.297	1.12E-48	0.268	1.20E-39	0.286	7.07E-45
AgeAccelHannum	0.225	4.12E-28	0.225	3.85E-28	0.192	1.05E-20
AgeAccelGrim	0.173	3.74E-17	0.167	5.80E-16	0.125	1.29E-09
DNAmADMAdjAge	0.121	4.58E-09	0.121	4.26E-09	0.076	2.62E-04
DNAmB2MAdjAge	0.189	4.69E-20	0.197	7.10E-22	0.088	2.26E-05
DNAmCystatinCAdjAge	0.119	8.71E-09	0.125	1.39E-09	0.031	1.29E-01
DNAmGDF15AdjAge	0.167	6.09E-16	0.181	1.58E-18	0.093	7.75E-06
DNAmLeptinAdjAge	-0.021	3.19E-01	-0.003	8.74E-01	-0.047	2.23E-02
DNAmPACKYRSAdjAge	0.166	7.99E-16	0.142	5.10E-12	0.172	7.31E-17
DNAmPAI1AdjAge	0.024	2.49E-01	0.056	6.70E-03	-0.053	1.12E-02
DNAmTIMP1AdjAge	0.070	7.04E-04	0.083	5.69E-05	0.021	3.06E-01
AgeAccelPheno	0.178	4.45E-18	0.168	3.52E-16	0.133	1.14E-10
DNAmTLAdjAge	-0.080	1.17E-04	-0.088	1.99E-05	-0.057	5.93E-03

DNAmAgeSkinBloodClockAdjAge	0.102	8.32E-07	0.127	7.26E-10	0.044	3.36E-02
Cell types						
CD8.naive	-0.072	5.20E-04	-0.071	5.91E-04	-0.040	5.38E-02
CD8pCD28nCD45RAn	0.085	3.90E-05	0.045	2.83E-02	0.104	5.07E-07
PlasmaBlast	-0.054	8.94E-03	-0.043	3.92E-02	-0.060	3.98E-03
CD4T	-0.146	1.68E-12	-0.138	2.08E-11	-0.096	3.89E-06
Gran	-0.075	2.72E-04	-0.106	3.10E-07	-0.017	4.15E-01

*Adjusted for age, sex, cell types.

Supplementary Table 20. FHS: Distribution of hype- and hypomethylated SEMs in relation to CpG island.

	Constantly Hypermethylated SEM *	Constantly Hypomethylated SEM **
**Open Sea**	8403	41533
**Island**	45807	5366
**N_Shelf**	711	5101
**N_Shore**	11205	6769
**S_Shelf**	619	4913
**S_Shore**	8483	5233

* Constantly hypermethylated SEMs were defined as DNA methylation mutations identified in more than 10 participants that were greater than three times the IQR above the upper quartile of a given CpG.
** Constantly hypomethylated SEMs were defined as DNA methylation mutations identified in more than 10 participants that were less than three times the IQR above the lower quartile of a given CpG.

Supplementary Table 21. FHS: Distribution of hype- and hypomethylated SEMs in relation to genomic region.

	Constantly Hypermethylated SEM *	Constantly Hypomethylated SEM **
**TSS1500**	11769	5841
**TSS200**	13399	1639
**5'UTR**	4536	3350
**1st Exon**	2160	628
**Gene body**	12757	26197
**3' UTR**	559	3442

* Constantly hypermethylated SEMs were defined as DNA methylation mutations identified in more than 10 participants that were greater than three times the IQR above the upper quartile of a given CpG.
** Constantly hypomethylated SEMs were defined as DNA methylation mutations identified in more than 10 participants that were less than three times the IQR above the lower quartile of a given CpG.

Supplementary Table 22. Association between Shannon entropy and age, AgeAccel, EML.

Outcome =Entropy *	FHS (n = 2326)		WHI (n= 2091)		JHS (n= 1734)		PEG 1 (n = 237)	
	Bicor r	P_value	Bicor r	P_value	Bicor r	P_value	Bicor r	P_value
Age	0.001	9.55E-01	0.068	2.01E-03	0.071	2.92E-03	0.117	7.30E-02
DNAm Age Acceleration								
AgeAccelHorvath	0.081	9.11E-05	0.160	1.76E-13	-0.039	1.02E-01	0.006	9.22E-01
IEAA	0.035	9.02E-02	0.131	1.70E-09	-0.052	3.16E-02	0.018	7.83E-01
EEAA (Unadjusted for cell types)	0.038	6.39E-02	0.090	3.60E-05	0.017	4.73E-01	0.123	5.93E-02
AgeAccelHannum	0.155	6.23E-14	0.136	4.60E-10	0.063	9.10E-03	0.096	1.41E-01
AgeAccelGrim	0.077	1.89E-04	0.228	3.86E-26	0.043	7.17E-02	0.164	1.14E-02
DNAmADMAdjAge	0.001	9.62E-01	0.205	2.80E-21	-0.022	3.49E-01	0.075	2.53E-01
DNAmB2MAdjAge	0.033	1.12E-01	0.057	9.76E-03	-0.019	4.19E-01	0.157	1.59E-02
DNAmCystatinCAdjAge	0.110	9.87E-08	0.422	5.62E-91	0.075	1.67E-03	0.200	1.93E-03
DNAmGDF15AdjAge	0.095	4.70E-06	0.226	1.53E-25	0.230	2.54E-22	0.076	2.45E-01
DNAmLeptinAdjAge	-0.023	2.68E-01	-0.060	6.11E-03	0.031	2.01E-01	-0.034	6.05E-01
DNAmPACKYRSAdjAge	0.091	1.17E-05	0.125	9.64E-09	0.054	2.46E-02	0.112	8.44E-02
DNAmPAI1AdjAge	-0.036	8.02E-02	0.009	6.68E-01	-0.007	7.78E-01	0.099	1.30E-01
DNAmTIMP1AdjAge	0.015	4.73E-01	-0.041	5.96E-02	-0.085	4.17E-04	-0.113	8.17E-02
AgeAccelPheno	0.079	1.35E-04	0.069	1.68E-03	-0.008	7.47E-01	-0.018	7.88E-01
DNAmTLAdjAge	0.002	9.36E-01	0.042	5.41E-02	0.017	4.78E-01	0.010	8.80E-01
DNAmAgeSkinBloodClockAdjAge	-0.025	2.35E-01	-0.063	4.18E-03	-0.001	9.81E-01	-0.072	2.67E-01
SEM	0.089	1.63E-05	0.294	6.87E-43	0.325	7.22E-44	0.281	1.10E-05

* Adjusted for Age, Sex, Race/ethnicity, Cell types.
